# Turn around to have a look? Spatial referencing in dorsal vs. frontal settings in cross-linguistic comparison

**DOI:** 10.3389/fpsyg.2015.01283

**Published:** 2015-09-02

**Authors:** Sieghard Beller, Henrik Singmann, Lisa Hüther, Andrea Bender

**Affiliations:** ^1^Department of Psychosocial Science, University of BergenBergen, Norway; ^2^Department of Psychology, University of ZürichZürich, Switzerland; ^3^Department of Psychology, University of FreiburgFreiburg, Germany

**Keywords:** spatial cognition, frames of reference (FoR), relative FoR variants, frontal vs. dorsal referencing, cross-linguistic comparison (German, US-English, Mandarin Chinese, Tongan), MPT modeling

## Abstract

When referring to an object in relation to another, speakers of many languages can adopt a relative frame of reference (FoR). Following Levinson (2003), this kind of FoR can be established by projecting an observer's perspective onto the ground object either by translation, reflection, or rotation. So far, research on spatial FoRs has largely ignored the extent of variation in which of these projections are preferred generally, and specifically what kind of FoR is established for spatial arrays in one's back. This may seem justified by assumptions on “natural” preferences: for reflection in frontal settings *(Canonical Encounter Hypothesis)*, and for converting dorsal into frontal situations by a turn of the observer before a reference is made *(Turn Hypothesis)*. We scrutinize these assumptions by comparing the FoRs adopted for small-scale, static spatial arrays by speakers of four languages (German, US-English, Mandarin Chinese, and Tongan). Addressing the problem of inherent ambiguities on the item level when assessing FoRs from spatial prepositions, we use a multinomial processing tree (MPT) model for estimating probabilities of referencing strategies across sets of items. Substantial differences in frontal settings, both between and within languages, disprove the Canonical Encounter Hypothesis—translation occurs as frequently as reflection across samples. In dorsal settings, in contrast, the same type of response dominates in all samples. We suggest that this response is produced by a *backward projection* of the observer's coordinate system in correspondence with the two main FoR preferences for frontal settings. However, none of these strategies involves a turn of the observer, thus also disproving the Turn Hypothesis. In conclusion, we discuss possible causes of the observed variability, explore links between the domains of space and time, and reflect the relation between language, communication, and culture.

## Introduction

When we are asked to locate an object in relation to another—for example, “where is the ball in relation to the box?”—we have to establish a coordinate system or a frame of reference (FoR) that allows us to derive a specific answer such as “The ball is in front and to the right of the box, from my point of view.” Spatial frames of reference can thus be regarded as cognitive tools that help us to interpret spatial relators in language and cognition (Bohnemeyer, 2011). A growing body of research indicates that, across languages, people differ in the frame of reference they preferentially adopt (overview in Majid et al., 2004). Variation has been documented especially with regard to which of the three *basic* types of FoRs is used: the *absolute* FoR derived from a superordinate field like the cardinal directions, the *intrinsic* FoR derived from an oriented object like a cat or a car, or the *relative* FoR derived from an observer (Senft, 1997; Pederson et al., 1998; Bennardo, 2002; Levinson, 2003; Dasen and Mishra, 2011; for alternative terminologies, see also Levinson, 2003, p. 26; Grabowski, 1999a,b; Talmy, 2000; O'Meara and Báez, 2011; Bohnemeyer and O'Meara, 2012).

When different types of FoRs are possible in a language, we typically observe a flexible referencing behavior depending on contextual factors of the situation and on characteristics of the objects involved (Bohnemeyer, 2011). Speakers of European languages, for instance, tend to adopt an absolute FoR in large-scale settings, but the intrinsic FoR or relative FoR in small-scale settings (Mishra et al., 2009). And when the ground object is oriented, intrinsic references increase at the cost of relative references (Schober, 1993, 1998; Carlson-Radvansky and Radvansky, 1996; Surtees et al., 2012), particularly when movement is involved (Hill, 1978; Levelt, 1984; Carroll, 1997; Grabowski and Miller, 2000).

Whether and which cognitive implications arise from such linguistic preferences is a matter of ongoing debate (Levinson et al., 2002; vs. Li and Gleitman, 2002; and see Haun et al., 2011; Li et al., 2011), but representations of space are now widely believed to be foundational to representations of more abstract domains such as time or number (e.g., Walsh, 2003; Dehaene and Brannon, 2010; Núñez and Cooperrider, 2013). Although the extent to which representations of space influence other domains remains controversial (Núñez et al., 2011, 2012; Bender et al., 2012a; Bender and Beller, 2014), the assumed links have invited research on intra- and cross-cultural variation in spatial representations.

**Figure 1:**
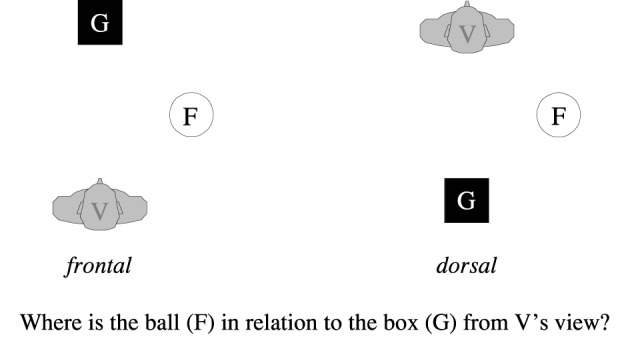
**A frontal and a dorsal configuration depicted from above (F, figure object; G, ground object; V, observer)**.

And yet, despite conceptual and empirical advances, the scientific landscape still contains considerably large patches of *terra incognita*. Barely any attention, for instance, has been devoted to the *variants* of the relative FoR (but see Bennardo, 2000; Levinson, 2003), despite the fact that variation has been known since Hill's (1978, 1982) comparison of English and Hausa. Even less is known about how people deal with spatial arrays that are not located in their visual field, but in their back (see Figure 1 for an example). Do they turn around—physically or mentally—thereby converting the dorsal into a frontal situation, and then employ the FoR variant they prefer for frontal settings? Disregarding these issues seems justified by default assumptions of “natural” preferences, as will be detailed below, but whether these assumptions are justified is empirically still an unanswered question.

### Variants of the relative FoR and the canonical encounter hypothesis

**Figure 2:**
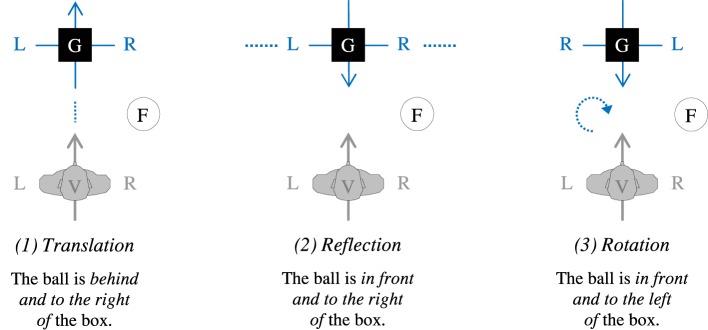
**Variants of the relative FoR for a frontal setting resulting from different projections of the coordinate system anchored in the observer V (Levinson, 2003)**. FRONT of a coordinate system is indicated by the tip of the arrow; L/R, left/right.

Frames of reference are used to localize a figure object F in reference to a ground object G. In contrast to the absolute and the intrinsic FoR, the relative FoR requires to do so from an observer's point of view V. To this end, the coordinate system, which is anchored in the observer (i.e., his or her FRONT/BACK and LEFT/RIGHT), is projected onto the ground object G (an idea already discussed by Bühler, 1934; see also Bühler, 1982, pp. 26–27). Following the terminology of Levinson (2003), this projection can be done in three ways: If the observer's coordinate system is *translated* into G, FRONT is assigned in gaze direction of V to the space beyond G, and BACK to the space between V and G. If it is *reflected* in G, FRONT is assigned to the space between V and G, and BACK to the space beyond G. In both cases, the assignment of LEFT and RIGHT is taken from the orientation of the observer. If it is *rotated* by 180° in G, FRONT is, again, assigned to the space between V and G, and BACK to the space beyond G, but the assignment of LEFT and RIGHT is now swapped (Figure 2). If adopting, for example, the reflection variant, the spatial array in Figure 2 would be described as “The ball is *in front and to the right of* the box”^1^.

Of the three types of projections, the reflection variant corresponds to the *Canonical Encounter Hypothesis* (Clark, 1973; Miller and Johnson-Laird, 1976; Grabowski and Miller, 2000; for a discussion see Grabowski, 1999b). Some scholars even presuppose it as the prototype of the relative FoR, for example, in experimental designs (as in Janzen et al., 2012) or in developmental tests (as in the *New Reynell Developmental Language Scales;* Edwards et al., 2011), which would actually require to acknowledge different kinds of FoRs.

Exceptions are only rarely acknowledged, although evidence for the use of the translation variant was reported already in the 1970s. Speakers of Hausa, for example, prefer translation (or “in-tandem prototype”) to refer to objects in their visual field, thus referring to an object further away in looking direction as “in front,” in contrast to speakers of English, who prefer reflection (or “mirror-image prototype”) in such contexts (Hill, 1978, 1982). But even among English speakers, the translation variant is not uncommon: Adults often adopt translation instead of reflection when movement is involved, thus referring to the object further away in moving direction as “in front” (Hill, 1978)—a tendency that can be explained by a different alignment of the coordinate system, namely with the direction of movement. And in a study of Harris and Strommen (1972), about 25% of English-speaking children preferred translation even in situations with static, non-oriented (and visible) objects. Only the observation of cross-linguistic variation is occasionally cited (e.g., Grabowski and Miller, 2000, p. 520), whereas the observation of intra-cultural variation has been largely ignored (Bender et al., 2012a).

Conceiving of FoR preferences as a matter of linguistic *convention* that has to be established among the speakers of a language, the different variants of the relative FoR need be regarded as equally possible (Levinson, 2003). What one might anticipate, then, is variability in relative FoRs across languages—and possibly also within languages—rather than a uniform pattern.

### Dorsal configurations and the turn hypothesis

When taking an observer's point of view, the distinction between frontal and dorsal is indispensable. And yet, research on FoRs has focused nearly exclusively on how people represent and describe relations between objects that are laid out *in front* of an observer. Hardly any attention has been devoted to the question of whether and how people describe relations between objects laid out *behind* them. Some researchers even argue that such dorsal referencing does not occur at all:

“Moreover, we presuppose that all entities involved are on the positive segment of the ordinate (i.e., from the origin's point of view), which is to say, that observers do not conceive of object relations in their back, but would rather turn around before” (Grabowski and Miller, 2000, p. 520, footnote 5; and see Grabowski, 1999b, p. 353; Grabowski and Weiß, 1996, p. 237).

This argument combines two claims, none of which has been empirically tested. The first claim holds that people *refrain* from conceiving of object relations in their back. While people may indeed prefer to talk about objects to which they have direct visual access, information on the situation in one's back nevertheless is accessed in various ways, and can be precisely described. “I heard a grunt behind me to the left, but couldn't see him” or “He backs up the car, pulls out behind me to the left, pulls into the spot on the left, backs up, goes behind me to the right, then leaves the subway” are just two of countless instances to be found on the internet that attest to this possibility.

**Figure 3:**
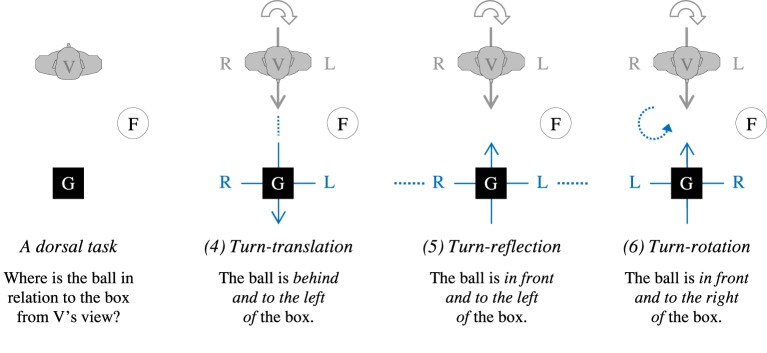
**Referring to objects in one's back according to the Turn Hypothesis: Turn by 180° and apply a FoR used in frontal settings (see Figure 2)**.

According to the second claim, when confronted with a dorsal situation, people should *turn around* to the objects in back of them, thereby converting the dorsal into a frontal situation, and then employ the FoR they usually adopt in the frontal case as shown in Figure 3 (*Turn Hypothesis*). This hypothesis thus includes a correspondence between frontal and dorsal situations with regard to which kind of projection is used: People with a preference for the translation variant of the relative FoR in frontal settings should adopt a turn-translation strategy in dorsal settings, those with a preference for the reflection variant should adopt a turn-reflection strategy, and those with a preference for the rotation variant should adopt a turn-rotation strategy.

The natural manner of such reorientation is bodily rotation—we literally “turn around”—but reorientation need not be performed *physically*. People may have observed the situation earlier and may have memorized it, or they may infer it from listening to sound signals or from observing it in a rear-view mirror. In these cases, people will construct a mental representation of the objects in their back, and might refer to them by turning around *mentally*. However, whether or not people actually perform such a turn, and if so, which FoR they subsequently adopt are questions requiring empirical investigation.

### Goals of the study

Our study aims at scrutinizing the two default assumptions on relative referencing: that *reflection* is the canonical variant of a relative FoR, and that dorsal configurations are turned into frontal ones by a (mental) *turn of the observer*. To this end, we investigate intra- and cross-linguistic variation in preferences for the variants of the relative FoR. As we are not primarily interested in communicative processes, but in how people understand and describe frontal vs. dorsal configurations, we restrict our study to small-scale and non-dynamic settings that do not involve an interlocutor. Two factors that might influence responses in such situations are included in the study: whether or not the ground object G is *oriented*, which might trigger intrinsic references instead of relative ones, and whether or not the entities are *animate*, which might strengthen intrinsic referencing, particularly for animates able to move.

#### A fundamental problem

Such an agenda, however, encounters a methodological problem gone unnoticed (or at least unreported) in previous work: Expanding the number of FoRs to be identified inevitably increases the number of ambiguous responses when assessing FoRs from spatial prepositions (or other spatial relators) used in verbal descriptions. For illustration, consider the following configuration: 
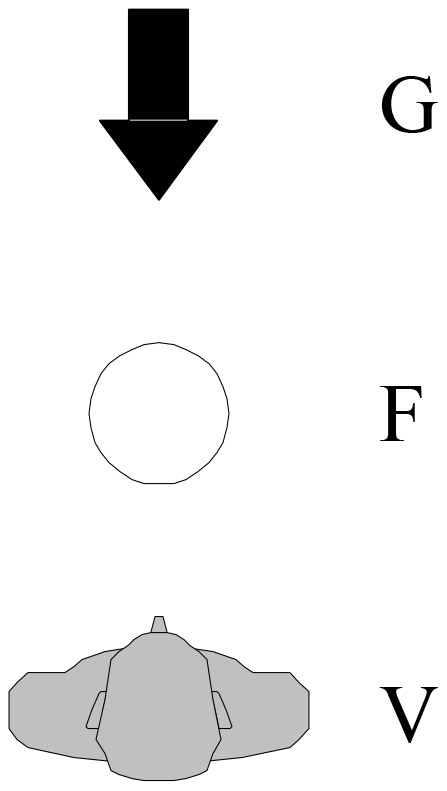


Assume a person refers to the circle as “in front of the arrow.” Which FoR does this reveal? Well, it could be the reflection or the rotation variant of the relative FoR (as projected from the orientation of the observer), it could also be the intrinsic FoR (anchored in the arrow), or it could be some not-yet-thought-of type of FoR.

The simplest strategy to deal with this problem is to omit all configurations that might produce such ambiguous responses. We will adopt this strategy, when providing a first, descriptive overview of our data. However, such an approach is not desirable because it restricts the types of configurations to be studied. Even if one were to consider only the three variants of the relative FoR depicted in Figure 2, trying to avoid ambiguous responses would systematically exclude all configurations in which F, G, and V are lined up as in the example in the left column, and all configurations in which they form a right angle (see **Figure 5A**). But, even if we accepted this constraint, we would still have no certainty that the ambiguity problem is solved, because we can only avoid ambiguities that result from reference patterns we already *know*. However, systematically excluding certain configurations on the basis of established reference patterns impairs the possibility to detect yet unidentified patterns.

In this article, we suggest a different approach: Instead of treating the ambiguity problem *on the item level* by excluding spatial configurations, we infer people's referencing strategies from a *set of items* by using a multinomial processing tree (MPT) model (Riefer and Batchelder, 1988). MPT modeling does not only allow us to utilize ambiguous and non-ambiguous items alike for estimating probabilities with which participants adopted specific FoRs, but also to test hypotheses on item-specific effects and cross-linguistic similarities and differences.

#### Selection of languages

As samples for our study, we chose native speakers of four languages—German, US-English, Mandarin Chinese, and Tongan—for three reasons.

First of all, from the scarcely available data on FoR preferences, interesting commonalities and differences emerged that seemed worth to be further explored. Speakers of the here investigated languages can, in principle, refer to spatial arrays by adopting any of the three *basic* types of FoRs: absolute, intrinsic, and relative (Levinson, 2003; Majid et al., 2004; Bennardo, 2009; Li and Zhang, 2009). With regard to which variant of the *relative* FoR people adopt, the following differences can be expected: Whereas German and English speakers are assumed to almost exclusively use reflection (Miller and Johnson-Laird, 1976; Grabowski and Weiß, 1996; Grabowski and Miller, 2000; Levinson et al., 2002), Tongan is one of the few languages for which habitual usage of translation has been reported (Bennardo, 2000; for two other cases in Polynesia and Africa, see Hill, 1978; Cablitz, 2006, respectively). For Mandarin Chinese, on the other hand—after all, the language with the most native speakers—we know nothing with regard to which variant of the relative FoR is preferred. The same is true for FoR preferences for dorsal settings in *all* of these languages. According to the Turn Hypothesis, we might hypothesize that German and English speakers prefer turn-reflection, while Tongan speakers might prefer turn-translation; for Chinese speakers, no hypothesis can be derived in advance. In case of an oriented ground object, English speakers appear to prefer the intrinsic FoR (Miller and Johnson-Laird, 1976; Cox, 1981; Abkarian, 1982), while German speakers do so only in some contexts (Grabowski and Miller, 2000).

The second reason for comparing these languages was that we had collected data on *temporal* frames of reference on all of them in a previous study (Bender et al., 2010). Collecting additional data on *spatial* FoRs would enable us to assess the nature and extent of cross-domain mapping between patterns of references in space and time that has remained elusive for too long (Núñez and Cooperrider, 2013; Bender and Beller, 2014). We will return to the issue of space-time mapping in the Discussion.

Finally, with the strategy of contrasting two more closely related languages (German and English are both Germanic languages) with two unrelated languages (Mandarin Chinese and Tongan) we hoped to gain some insights into the level on which differences in referencing strategies emerge: on the level of the vocabulary (if relatedness is important) or on the pragmatic level established among the community of speakers.

## Frontal and dorsal references in cross-linguistic comparison

The study aims at examining which preferences speakers of different languages (German, US-English, Mandarin Chinese, and Tongan) have for the variants of the relative FoR in frontal and dorsal settings.

### Method

Spatial references were assessed with a paper-pencil questionnaire. As in the examples in Figure 1, participants were asked to take the perspective of a depicted observer and to describe, from this perspective, object arrays in front of this person (*frontal* condition) or in back of this person (*dorsal* condition).

#### Participants

The German sample consisted of 137 participants (101 female) from the Freiburg area, mostly students from Freiburg University (mean age 24.9 years; *SD* = 7.0; *range*: 18–58 years), 69 in the frontal and 68 in the dorsal condition. The US sample consisted of 137 participants (88 female) from the Pennsylvania area, mostly students from the Pennsylvania State University (mean age 21.1 years; *SD* = 4.3; *range*: 18–54 years), 67 in the frontal and 70 in the dorsal condition. The Chinese sample consisted of 70 students (21 female) from Tongji University in Shanghai (mean age 20.5 years; *SD* = 2.1; *range*: 16–24 years), 36 in the frontal and 34 in the dorsal condition. Finally, the Tongan sample consisted of 116 students (68 female) from Ha'apai High School (mean age 16.4 years; *SD* = 1.1; *range*: 14–19 years), 56 in the frontal and 60 in the dorsal condition.

#### Materials

All items required participants to adopt the perspective of an observer. The observer's gaze direction was always aligned with the participant's gaze direction. The objects to be related were located either in the visual field of the observer (*frontal* condition) or in the observer's back (*dorsal* condition).

**Figure 4:**
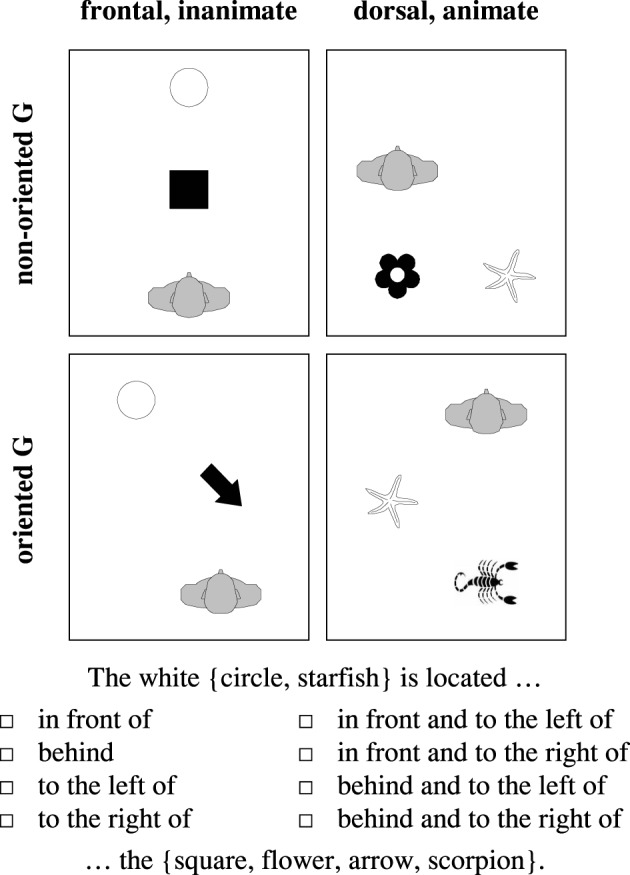
**Four example items**.

Twelve analogous configurations were used in each condition: six with an oriented ground object (three depicting inanimate objects, three depicting living beings) and six with a non-oriented ground object (again three depicting inanimate objects and three depicting living beings). Participants were asked to indicate the relation between figure F and ground object G from the viewpoint V of the depicted observer by choosing one of eight options: *in front of, behind, to the left of, to the right of, in front and to the left of, in front and to the right of, behind and to the left of, and behind and to the right of*. A selection of items is presented in Figure 4. The instructions and the complete set of items are provided for each of the four languages in the Supplementary Material (Sections 1 and 2).

**Figure 5:**
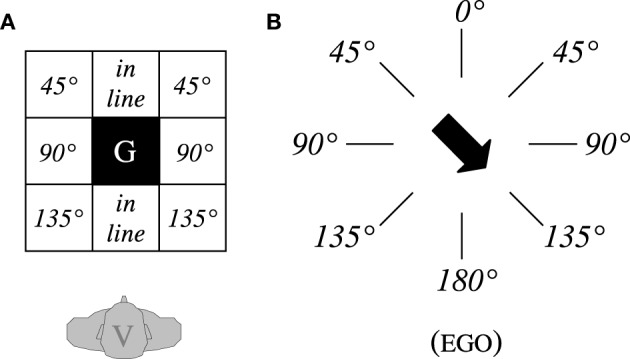
**(A)** Possible positions for the figure F in relation to the ground object G. **(B)** Directions of an oriented G (angular deviation from EGO's gaze direction).

Three types of configurations were used, enabling us to elicit the full range of response options: F was either *in line* with V and G, or it was displaced from this line *laterally by 90*°, or *diagonally by 45*° or *135*° (Figure 5A). These configurations differ in their requirements for responding according to a relative FoR and thus in their *configurational* complexity (see Grabowski, 1999b, pp. 360–361). If F is in line with V and G, the answer requires a FRONT/BACK assignment only (“low” complexity = 1). If F is displaced by 90°, the answer requires a LEFT/RIGHT assignment, which presupposes a FRONT/BACK assignment (“medium” complexity = 2). And if F is displaced by 45 or 135° in either direction, the answer requires both a FRONT/BACK and a LEFT/RIGHT assignment (“high” complexity = 3).

Moreover, if the ground object G affords adoption of the intrinsic FoR, the ease with which this FoR is applied may depend on the orientation of G, in other words: on the angle by which the coordinate system of the referencing individual (EGO) must be rotated in order to map it onto G (Figure 5B). Two aspects contribute to this *mapping difficulty:* whether or not the front of G points roughly in gaze direction of ego (angles 0° or 45°: low difficulty *d* = 1; 90° or more: higher difficulty *d* = 2), and whether or not the response requires to determine LEFT/RIGHT (no: difficulty *d* = 1; yes: *d* = 2). Multiplicatively combining the two criteria defines three classes of difficulties; *d* = {1, 2, 4}. The mapping difficulties and configurational complexities of all 24 items are provided in the Supplementary Material (Section 2).

#### Design and procedure

In all countries, the tasks were part of a larger survey on spatial references implemented as paper-pencil questionnaire. All materials were presented in the participants' native language (German, US-English, Mandarin Chinese, or Tongan); they had been translated by bilinguals and double checked in repeated re-translation sequences.

The twelve items of each condition (frontal and dorsal) were arranged in one of two orders: The first one started with the six non-oriented items (in random order) and then proceeded with the oriented items (also in random order); the second order was the exact reversal and thus started with the oriented items. The eight response options were always in the same order.

Participants were tested indoors, either individually or in small groups. Each individual was randomly assigned to one of four questionnaire versions (frontal condition with either first or second order of items, and dorsal condition with either first or second order of items).

Participation was voluntary, and informed consent was obtained from all participants prior to data collection in accordance with the ethical guidelines of the respective institution^2^.

### Results

The results are presented in three sections. First, we provide a descriptive overview of the data. Then, we use an MPT model to test item-specific effects and cross-linguistic similarities and differences. Finally, we inspect individual consistency in FoR choice across the item set.

#### Descriptive overview

In order to provide a first, descriptive overview of the data, we determined for each type or variant of FoR (a) in which of the items this FoR could be identified unambiguously, and (b) how frequently this type or variant of FoR was applied among these items. In the frontal condition, we distinguished between the three variants of the relative FoR (translation, reflection, and rotation; Figure 2), and the intrinsic FoR for the items with an oriented G. In the dorsal condition, we distinguished between three variants of the relative FoR according to the Turn Hypothesis (turn-translation, turn-reflection, and turn-rotation; Figure 3), and the intrinsic FoR for items with an oriented G. Responses that did not result from any of these variants were classified as “unknown” types of references.

Separately for each of the four sets of six items (frontal non-oriented vs. oriented, dorsal non-oriented vs. oriented), we excluded all participants with more than one missing response. This resulted in 13 exclusions from the total of 456 participants. As the factor *animacy* did not make any difference (neither descriptively nor later in the MPT analysis), animate and inanimate items were pooled. The results are presented in Table 1 (including final sample sizes for each item set).

**Table 1:** **Frequencies of FoR variants (in %) calculated from unambiguous items**.

**FoR (number of items)**	**German**		**English**		**Chinese**		**Tongan**	
	**Non-oriented**	**Oriented**	**Non-oriented**	**Oriented**	**Non-oriented**	**Oriented**	**Non-oriented**	**Oriented**
**FRONTAL ITEMS**								
Intrinsic (n.a._non−*oriented*_; 4_oriented_)	—	1.8	—	6.3	—	17.9	—	16.7
Translation (4_non−*oriented*_; 3_oriented_)	8.7	9.7	24.6	22.9	**41.7**	**43.8**	**54.8**	**38.5**
Reflection (2_non−*oriented*_; 3_oriented_)	**79.7**	**77.3**	**63.6**	**62.2**	20.8	15.2	11.5	16.8
Rotation (4_non−*oriented*_; 4_oriented_)	0.4	2.2	1.5	0.4	12.5	2.9	10.1	7.4
Unknown (6_non−*oriented*_; 6_oriented_)	5.1	4.6	4.0	5.0	17.6	17.1	25.3	24.7
*N* of sample	69		66	67	36	35	52	54
**DORSAL ITEMS**								
Intrinsic (n.a._non−*oriented*_; 4_oriented_)	—	1.8	—	14.3	—	23.5	—	25.5
Turn-translation (4_non−*oriented*_; 3_oriented_)	2.9	1.0	3.6	0.0	8.9	1.0	10.2	1.7
Turn-reflection (2_non−*oriented*_; 3_oriented_)	7.4	8.8	0.0	0.5	3.4	5.1	3.4	7.4
Turn-rotation (4_non−*oriented*_; 4_oriented_)	**83.5**	**84.6**	**87.4**	**75.0**	**76.4**	**61.4**	**54.9**	**37.0**
Unknown (6_non−*oriented*_; 6_oriented_)	4.7	3.7	6.9	7.9	12.0	7.1	31.4	26.6
*N* of sample	68		70		32	33	59	

*Number of unambiguous items, on which the analysis is based, are given in brackets for the sets of non-oriented and oriented items. Percentages need not add up to 100; n.a., not applicable; modal response printed in bold face*.

The proportion of responses that could be attributed to one of the FoR variants under scrutiny was generally high: 87.3% on average across all languages and tasks (corresponding to 12.7% “unknown” types of references). Looking at the modal responses in the frontal condition, the data suggest a preference for reflection in German and English, and for translation in Chinese and Tongan. In the dorsal condition, turn-rotation dominated in all four languages alike although to different proportions. In tasks with an oriented G, the intrinsic FoR was adopted to some degree by the English speaking participants, and to a larger degree by the Chinese and Tongan ones.

At this point, we should address one methodological concern: All configurations were presented as two-dimensional (2D) sketches from a bird's eye view and, thus, clearly differ from real-world spatial situations. Associated with this presentation are two questions: First, did the 2D sketches as such induce some kind of bias? And second, did our participants in fact adopt the point of view of the observer depicted in the scene?

With regard to the first question, the frontal items are indicative. Here, the descriptive results replicate the findings for the relative FoR variants obtained with other methods: a preference for reflection among German speakers (e.g., Grabowski and Miller, 2000; Beller et al., in press), mainly reflection, but also translation among English speakers (e.g., Hill, 1982; Grabowski and Miller, 2000), and a preference for translation among Tongan speakers (Bennardo, 2000). We therefore believe that our 2D sketches did not induce substantial biases.

With regard to the second question on perspective taking, the frontal items are not indicative. As the depicted observer was always looking in the same direction as the participant (aligned perspectives), the very same responses result regardless of whether participants project their own coordinate system onto the ground object G or the coordinate system anchored in the observer. The dorsal items, however, are indicative. Here, making a reference from the participant's point of view, while disregarding the depicted observer, would have led to different responses—and in this case more “unknown” types of references—at least for those participants with a preference for the reflection variant of the relative FoR. We therefore consider it safe to assume that the gross of our participants considered the observer's perspective.

The descriptive findings already address the two main hypotheses under scrutiny and clearly refute them: The reflection variant of the relative FoR does not generally prevail in frontal configurations (as suggested by the *Canonical Encounter Hypothesis*), and the majority of participants did obviously not combine a turn of the observer with their preferred *frontal* variant (reflection or translation) in dorsal configurations (as suggested by the *Turn Hypothesis*). A suggestion for why they chose the turn-rotation response instead will be presented in the Discussion.

For this overview, we restricted the considered items in order to deal with the general problem that not every FoR can be unambiguously assessed on every item, but in doing so, we of course lost information. A more elegant way is provided by multinomial processing tree modeling. This technique enables us not only to estimate probabilities of the referencing strategies from *all* items, but also to consider the influence of the item-specific factors *configurational complexity, mapping difficulty*, and *animacy*, and to test hypotheses on cross-linguistic similarities and differences.

#### An MPT-model of frontal and dorsal references

MPT models are cognitive measurement models that describe categorical data by a set of latent cognitive states (for reviews see Batchelder and Riefer, 1999; Erdfelder et al., 2009). Each cognitive state is represented by a parameter that reflects the probability with which the state is reached. The cognitive states are assumed to unfold like a tree from the “root node” to the “leaves” (the response categories) by binary branching^3^. Thereby, the problem of ambiguity in FoR assessment on the item level can be quite simply addressed: Unambiguous responses result only from one path in a tree (i.e., from one specific FoR), whereas ambiguous responses result from different paths (i.e., from different FoRs). The probability of any response category can then be calculated by multiplying the parameters along one path from the root to the leaf. In case multiple branches lead to the same response category, the individual products are summed. Ambiguities can be resolved across a set of items, as long as the ambiguity does not concern all items alike. The model thus assumes that the probability with which a specific FoR is instantiated is identical across items and that this probability is independent from whether or not an item is ambiguous. A violation of this assumption would result in considerable model misfit.

##### The full model

**Figure 6:**
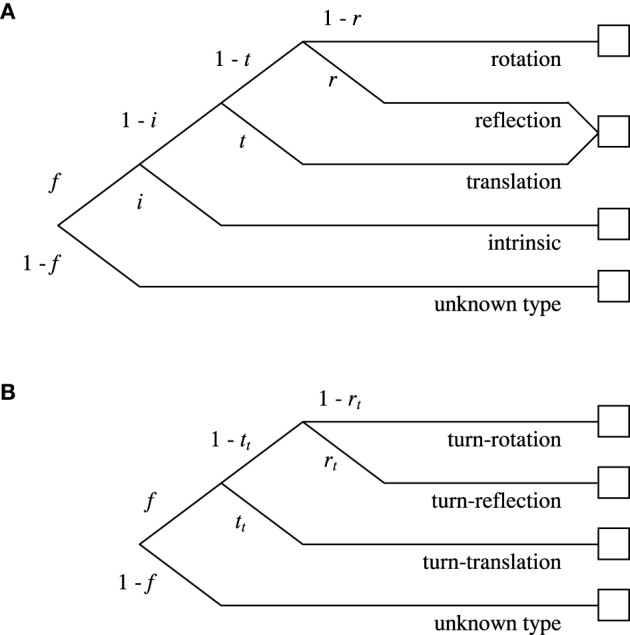
**Two example trees and their parameters**. **(A)** Process model for an *ambiguous* frontal item with oriented G. **(B)** Process model for an *unambiguous* dorsal item with non-oriented G. Trees are traversed from left to right. Each node represents a latent cognitive state with the edges to each node containing the parameter leading to this node. The squares on the right represent the response categories. The complete list of processing trees is given in Section 2 of the Supplementary Material.

The full model consists of one tree per item (i.e., 24 trees in total) and 44 parameters per language. For each item, we first distinguish whether or not a person responds with an identifiable FoR (represented by the parameters *f* vs. 1 – *f*). As not responding with an identified FoR might happen independently for each item, for example due to a not covered type of FoR, some kind of error, or guessing, the full model comprises 24 *f* parameters per language, one for each item. For items with an oriented ground object, we then distinguish whether the intrinsic FoR or a variant of the relative FoR is used (*i* vs. 1 – *i*). As the decision to adopt the intrinsic FoR might depend on item-specific characteristics, for example on the mapping difficulty or on whether or not the objects are animate, the full model comprises 12 *i* parameters per language, one for each item with an oriented G. To represent adoption of the different variants of the relative FoRs, different sets of parameters are used for the frontal and the dorsal items: In the frontal case, we distinguish whether the translation variant or a different variant is adopted (*t* vs. 1 – *t*), and whether this is reflection or rotation (*r* vs. 1 – *r*). In the dorsal case, we distinguish whether the turn-translation variant or a different variant is adopted (*t*_*t*_ vs. 1 – *t*_*t*_), and whether this is turn-reflection or turn-rotation (*r*_*t*_ vs. 1 – *r*_*t*_). Each of the four parameters *t, r, t*_*t*_, and *r*_*t*_ is implemented in two versions in order to be able to model that the ratios of relative references might depend on whether or not items contain an oriented G. Figure 6 shows two example trees, one for an ambiguous frontal item and one for an unambiguous dorsal item. The complete set of trees is provided in Section 2 of the Supplementary Material.

The response categories were formed separately for each item (in each language) from the eight possible responses; all responses that were not indicative for any of the FoRs under scrutiny were summed up in the category “unknown type of reference.” In total, 90 response categories per language entered the analysis, corresponding to 66 independent data points. The model parameters were estimated from the frequencies of the response categories aggregated across participants by using maximum likelihood estimation with MPTinR (Singmann and Kellen, 2013). Summed across the languages, the full model revealed a good overall fit; *G*^2^(88) = 80.1; *p* = 0.71; and in none of the languages did the model provide a significant misfit; all *G*^2^(22) < 27.2; *p*>0.20. These values indicate that our modeling assumptions are by and large compatible with the data. The complete list of parameter estimates is reported in Section 3.1 of the Supplementary Material.

##### Model selection: testing for item-specific effects and language differences

For a more in-depth analysis of the data, we then probed a selection of restricted models in order to find the model with the best balance between model fit and parsimony. This *model selection* process (Zucchini, 2000) was performed in four steps. In steps 1 and 2 we tested whether all item-specific parameters are necessary, or whether the data can also be accounted for with a more parsimonious version of the model. For steps 3 and 4, we took the most parsimonious model from step 2 to test specific hypotheses on cross-linguistic similarities and differences. Step 4 provides us with a final model from which we then calculated the prevalence of the different variants of the relative FoR in our samples.

Model selection is achieved by combining a model's goodness of fit with a penalty based on its complexity. While the classical indices *AIC* and *BIC* (e.g., Burnham and Anderson, 2002) use the number of parameters as a proxy for complexity, we used the *Fisher Information Approximation (FIA)* assessing complexity by estimating a model's ability “to compress data.” Each FIA calculation is based on 200,000 Monte Carlo samples (following Wu et al., 2010a,b), and all FIA penalties are logically consistent, that is, nested models always have lower penalties than the superordinate model(s) (Navarro, 2004). The complete model selection analysis is reported in the Supplementary Material, Section 3.2.

##### Step 1: item-specific effects (identified FoRs and intrinsic FoRs)

With one *f* parameter for each item and one *i* parameter for each oriented item, the full model allows for item-specific rates of identified FoRs (*f*) and, among these, of intrinsic FoRs (*i*) in each language. In step 1 of the model selection process, we checked whether this item-specificity is necessary or whether there is evidence for more general tendencies. We hypothesized that the frequency of identified FoRs may depend on an item's *configurational complexity*, and that intrinsic referencing may depend on factors related to the orientation of the ground object (i.e., *mapping difficulty* and *animacy)*.

##### Restrictions for *f*

Five sets of parameter restrictions were considered: (1) The rates of identified FoRs might vary freely; in this case, all 24 *f* parameters are necessary to model the data from each language (model *f: free*). (2) On the other hand, these rates might be item-independent; in this case, one *f* parameter per language suffices *(f: all equal)*. (3) If the rates of identified FoRs depend on the three levels of configurational complexity alone, then three *f* parameters per language are necessary *(f: global complexity)*. Otherwise, these rates might depend (4) additionally on the frontal vs. dorsal perspective, which then implies two sets of three *f* parameters per language *(f: perspectival complexity)*, or (5) additionally on the orientation of the ground object, which then implies four sets of three *f* parameters per language *(f: local complexity)*.

##### Restrictions for *i*

Six sets of parameter restrictions were considered: The tendency to use the intrinsic FoR might be (1) item-specific (*i: free;* twelve *i* parameters per language) or (2) item-independent (*i: all equal;* one *i* parameter per language). With regard to mapping difficulty, the tendency to use the intrinsic FoR might depend (3) on the three difficulty levels alone (*i: global mapping;* three *i* parameters per language) or (4) additionally on the frontal vs. dorsal perspective (*i: perspectival mapping;* two sets of three *i* parameters per language). With regard to animacy, the tendency to use the intrinsic FoR might depend (5) on animacy alone (*i: global animacy;* two *i* parameters per language, one for living beings and one for inanimate objects) or (6) additionally on the frontal vs. dorsal perspective (*i: perspectival animacy;* two sets of two *i* parameters per language).

##### FIA results

We compared 30 models per languages for the four languages simultaneously, but allowing for language-specific fits: each of the five restrictions for the parameter *f* combined with each of the six restrictions for *i*. The model with the overall best performance was the model *f: global complexity* & *i: all equal* with 12 parameters per language; *FIA* = 272.9; *G*^2^(216) = 293.6; *p* < 0.001^4^. According to this model, the rates of identified FoRs depend on the complexity of the basic configuration, equally for all types of items (one *f* parameter per language for each complexity level), while the tendency to use the intrinsic FoR is item-independent (one *i* parameter per language).

Across the board, the *f* parameters varied as predicted across the three types of configurations, both for frontal and dorsal items. Tasks that required only a FRONT/BACK assignment (“in line”) showed higher rates *f* of identified FoRs on average than tasks that required a LEFT/RIGHT assignment or both (displaced by 90° or by 45°/135°; see Table 2A). The rate of intrinsic references was similarly high for frontal and dorsal items—which is consistent with the assumption that for intrinsic references, the position of the observer is irrelevant—but differed between languages (Table 2B). Surprisingly, neither mapping difficulty nor animacy was necessary to explain the data.

**Table 2:** **Parameter estimates (printed in bold face) and 95% confidence intervals of the final model**.

**Item type**	**Parameter**	**German**	**English**	**Chinese**	**Tongan**
**(A)** All items	*f_1_* (in line)	**1.0** (0.99; 1.0)		**0.88** (0.83; 0.92)	**0.86** (0.82; 0.90)
	*f_2_* (90°)	**0.97** (0.96; 0.98)		**0.88** (0.83; 0.91)	**0.66** (0.62; 0.70)
	*f_3_* (45°/135°)	**0.90** (0.89; 0.92)		**0.84** (0.81; 0.88)	**0.70** (0.67; 0.74)
**(B)** Items with oriented G	*i*	**0.02** (0.01; 0.03)	**0.11** (0.09; 0.14)	**0.27** (0.24; 0.31)	
**(C)** Frontal items	*t*	**0.09** (0.07; 0.12)	**0.25** (0.22; 0.29)	**0.64** (0.60; 0.68)	
	*r*	**0.99** (0.98; 0.99)		**0.66** (0.59; 0.74)	
**(D)** Dorsal items	*t_*t*_*	**0.03** (0.02; 0.04)		**0.09** (0.07; 0.11)	
	*r_*t*_*	**0.09** (0.06; 0.11)	**0.01** (0.00; 0.02)	**0.08** (0.05; 0.11)	

*Confidence intervals are based on 10,000 non-parametric bootstrap samples. Shared values represent parameters that could be set equal across items or between languages (according to the model selection)*.

##### Step 2: item-specific effects (relative FoRs)

The next step started from the best model from step 1 and tested whether or not the decision for a specific variant of the relative FoR is independent of whether or not the ground object is oriented.

##### Restrictions for *t, r, t*_*t*_, and *r*_*t*_

For each of these parameters, two models were defined: (1) one with only one parameter per language, representing both oriented and non-oriented items *(global)*, and (2) one with two parameter versions per language *(local)*.

##### FIA results

We compared 16 models (2^4^ combinations of restrictions) per language for the four languages simultaneously, again allowing for language-specific fits. The model with the overall best performance contained 8 parameters per language; *FIA* = 262.5. It identified all four parameters *t, r, t*_*t*_, and *r*_*t*_ as global.

In other words: The proportions of the different variants of the relative FoRs can be assumed to be equal for oriented and non-oriented items within each language (Table 2C,D).

##### Steps 3 and 4: cross-linguistic similarities and differences

So far, the model was fitted for each language individually. In the next two steps, we tested the data for similarities and differences between languages. To this end, we fitted the data of all languages simultaneously and tested different restrictions of parameters *across* languages.

##### Restrictions for differences between languages

For each parameter, *f, i, t, r, t*_*t*_, and *r*_*t*_, we tested five restrictions: (1) The parameter might vary freely between the four languages, indicating that the languages differ in frequencies of the respective responses (*all languages different;* four parameters, one for each language), or (2) the frequencies might be equal across languages so that one parameter suffices to model the data of all languages *(all languages equal)*. In addition, we tested whether the parameter in question can be set equal (3) for German and English, the two Germanic languages with a preference for reflection in frontal settings (*germanic equal;* three parameters: Germanic, Chinese, and Tongan), (4) for Chinese and Tongan, the two non-Germanic languages with a preference for translation in frontal settings (*non-germanic equal;* three parameters: German, English, and non-Germanic), (5) or both (*germanic equal + non-germanic equal;* two parameters: Germanic and Non-Germanic).

##### Step 3: language differences (identified FoRs and intrinsic FoRs)

First, we tested the probabilities of identified FoRs *(f)* and, among these, of the intrinsic FoR *(i)* for language differences.

##### FIA results

We considered 25 models: each of the five restrictions applied to the parameter *f* combined with each of the five restrictions applied to *i*. The model with the best performance had 28 parameters; *FIA* = 258.5. It identified *f* as *germanic equal* and *i* as *non-germanic equal*. In other words: The rate of identified FoRs *f* was similar for German and English, but differed for Chinese and Tongan both from that of the Germanic languages and from each other. Conversely, the tendency *i* to use the intrinsic FoR was similar for Chinese and Tongan, but differed for German and English both from that of the non-Germanic languages and from each other.

With regard to the rate of identified FoRs *f*, variation across the three types of configurations (in line vs. displaced by 90° or 45°/135°) was least among the Chinese participants (*range:* 0.04), larger among German and US participants (*range:* 0.10), and largest among the Tongan participants (*range:* 0.20; Table 2A). The rate of intrinsic references *i* varied considerably across languages; it was lowest among the German participants, larger for the US participants and largest among the Chinese and Tongan participants (Table 2B).

##### Step 4: language differences (relative FoRs)

The final step of the analysis was carried out separately for the frontal and dorsal items. In both cases, we started from the best model of step 3, and tested the parameters for the variants of the relative FoR (*t* and *r; t*_*t*_ and *r*_*t*_) for language differences.

##### FIA results for the frontal items

We considered 25 models: each of the five restrictions applied to the parameter *t* combined with each of the five restrictions applied to *r*. The number of parameters could again be reduced compared to the best model from step 3: The final model for the frontal data had 25 parameters; *FIA* = 254.9. It identified *t* as *non-germanic equal* and *r* as *germanic equal + non-germanic equal*. In other words: The proportion of translation *t* was similar for Chinese and Tongan, but differed for German and English both from that of the non-Germanic languages and from each other, whereas the ratio between reflection and rotation (as represented by *r*) was similar between Chinese and Tongan, and similar between German and English (Table 2C).

##### FIA results for the dorsal items

Again, we considered 25 models: each of the five restrictions applied to the parameter *t*_*t*_ combined with each of the five restrictions applied to the parameter *r*_*t*_. Compared to the best model from step 3, the number of parameters could again be further reduced: The final model for the dorsal data had 25 parameters; *FIA* = 255.3. It identified *t*_*t*_ as *germanic equal + non-germanic equal* and *r*_*t*_ as *non-germanic equal*. In other words: The proportion of turn-translation *t*_*t*_ was similar for German and English and similar for Chinese and Tongan, whereas the ratio between turn-reflection and turn-rotation (as represented by *r*_*t*_) was similar for Chinese and Tongan, but differed for German and English, both from that of the non-Germanic languages and from each other (Table 2D).

##### Final model and probabilities of the relative FoRs

The overall *final model* combined the restrictions of the best models from step 4 for the frontal and dorsal items. This model had 22 parameters; FIA = 251.8; *G*^2^(242) = 366.3; *p* < 0.001. The parameter estimates are shown in Table 2; the estimates of the variants of the relative FoRs in Table 3.

**Table 3:** **Probabilities for the variants of the relative FoR (and 95% confidence intervals), given that a relative FoR is adopted**.

**FoR**	**German**	**English**	**Chinese**	**Tongan**
**(A) FRONTAL ITEMS**				
Translation	0.09 (0.07; 0.12)	0.25 (0.22; 0.29)	**0.64 (0.60; 0.68)**	
Reflection	**0.89 (0.87; 0.92)**	**0.73 (0.69; 0.77)**	0.24 (0.20; 0.28)	
Rotation	0.01 (0.01; 0.02)	0.01 (0.01; 0.02)	0.12 (0.09; 0.15)	
**(B) DORSAL ITEMS**				
Turn-translation	0.03 (0.02; 0.04)		0.09 (0.07; 0.11)	
Turn-reflection	0.08 (0.06; 0.11)	0.01 (0.00; 0.02)	0.07 (0.05; 0.10)	
Turn-rotation	**0.89 (0.86; 0.91)**	**0.97 (0.95; 0.98)**	**0.84 (0.80; 0.87)**	

*Probabilities are computed from the parameters of the final model (Table 2). For frontal items: translation = t; reflection = (1 - t) × r; rotation = (1 - t) × (1 - r). For dorsal items: turn-translation = t_t_; turn-reflection = (1 - t_t_) × r_t_; turn-rotation = (1 - t_t_) × (1 - r_t_). Shared values represent probabilities that could be set equal between countries. Confidence intervals are based on 10,000 non-parametric bootstrap samples. Modal response printed in bold face*.

The frontal data revealed strong differences between languages with regard to the variants of the relative FoR (Table 3A). For German, reflection by far prevailed (89% of all participants who applied a relative FoR) over the second most frequent FoR, translation (9%). For English, reflection also dominated (73%), but translation was more prominent (25%) than in German. In these two languages, rotation was nearly absent. Finally, for Chinese and Tongan, all three FoRs were observed with translation being dominant (64%). By contrast, language differences were small for the dorsal data (Table 3B). Among all participants who applied a relative FoR, the turn-rotation response by far prevailed (between 84 and 97%). This choice and its homogeneity are not only surprising because rotation is very rarely used as projection for frontal settings, but also when contrasted with the substantial cross-linguistic differences in strategies for frontal tasks. We will return to this puzzling finding in the Discussion.

##### Model fit

While the full model with all 176 parameters fitted the data quite well according to the summed *G*^2^ statistics, the *G*^2^ statistics also indicated a significant misfit (*p* < 0.001) for the best performing model in each of the model selection steps. One may therefore wonder whether or not the results of the different steps and particularly the language comparisons are sound. To answer this question, it is important to remember that the goal of the model selection process is different from the goal underlying the use of the *G*^2^ statistics, which provides an assessment of descriptive adequacy only. In contrast, model selection is concerned with choosing from a set of candidate models the one model that best captures the regularities in the data. The conclusions drawn from a model that provides a good approximation of the regularities can be more validly generalized from observed data to yet unobserved data (e.g., Wu et al., 2010a). Relating these considerations to our findings, we can conclude: First, the full model provided an adequate account of the data, implying that our modeling assumptions are empirically adequate. And second, the fact that the eventually selected models seemed to misfit bears no consequences on the conclusions. To the contrary, it avoids overfitting by focussing on the relevant characteristics present in the data.

#### Individual consistency in FoR choice

Does the variety of responses that we observed on the aggregate level result from intra-individually varying, task-specific references or from individually stable, but inter-individually different preferences for a particular FoR? In order to answer this question, we determined whether participants adopted a particular FoR consistently and, if so, which one.

To this end, we counted for each participant and in each of the four sets of items (frontal non-oriented vs. oriented, dorsal non-oriented vs. oriented) how many responses were consistent with the same one of the FoRs, whether ambiguously or not. For example, if four of a participant's responses to the six frontal oriented items were consistent with reflection, two with translation, and two were characterized as unknown types of references, consistency would be 66.7% for reflection and 33.3% for translation. The *maximum* of these values (here: 66.7%) indicates the FoR adopted most often, and how often it could be diagnosed across the items of the respective set, and may thus serve as an estimate of an individual's consistency in FoR adoption. Mean consistency values are displayed in Table 4.

**Table 4:** **Individual consistency in FoR adoption (in % of items)**.

**Type of item**	**German**	**English**	**Chinese**	**Tongan**
Frontal, non-oriented G	92.3 (69)	91.9 (66)	75.0 (36)	67.0 (52)
Frontal, oriented G	91.5 (69)	87.3 (67)	74.3 (35)	59.0 (54)
Dorsal, non-oriented G	92.2 (68)	89.0 (70)	78.1 (32)	61.6 (59)
Dorsal, oriented G	92.6 (68)	81.4 (70)	82.3 (33)	55.1 (59)

*The values represent the average frequency by which an individual's most frequently adopted FoR was diagnosed in the respective item set. Numbers in parentheses represent the n of participants*.

In general, responses were intra-individually quite consistent, with a mean value of 79.4% on average across the four languages. In other words: Participants adopted their individually preferred FoR in 4.74 of 6 items of a block. An analysis of variance of the consistency values as dependent variable with one within-subject factor *item type* (oriented vs. non-oriented) and the two between-subjects factors *perspective* (frontal vs. dorsal) and *language* revealed a main effect *language* [*F*_(3, 443)_ = 61.91; *p* < 0.001; η^2^ = 0.295], a main effect *item type* [*F*_(1, 443)_ = 9.83; *p* = 0.002; η^2^ = 0.022], and an interaction *language* × *item type* [*F*_(3, 443)_ = 5.24; *p* = 0.001; η^2^ = 0.034]. *Post-hoc* analyses indicated that German and English speakers did not differ in consistency (92.2 and 87.4%), and that both Chinese and Tongan speakers (77.2 and 60.9%) differed from the speakers of the three other languages; *p* < 0.05; Bonferroni corrected. Consistency was slightly lower for the items with an oriented ground object G (78.0%) than for the items with a non-oriented G (80.9%), and this difference varies between the four countries, as reflected in the interaction. Again, there were no effects of the two perspectives: Frontal items (79.9%) and dorsal items (79.0%) were answered with nearly the same consistency. Taken together, these findings suggest that only the possibility of adopting an additional FoR (here: intrinsic) is a source of inconsistency, but not the atypical dorsal situation.

Next, we identified each participant's preferred FoR as the one response category that was assessed (a) more often than all others and (b) in at least four out of the six items of a block (i.e., with a consistency of at least 66.7%). Participants' preferred FoR variants are presented in Table 5. The individually preferred FoRs reflect the aggregated data from Table 1 quite nicely: If the ground object was oriented, some participants consistently adopted the intrinsic FoR. With regard to relative FoRs, translation and reflection were preferred in the frontal condition and turn-rotation in the dorsal condition. Finally, in China and Tonga, the proportion of participants with no clear preference for any FoR variant was substantially higher than in Germany and in the US.

**Table 5:** **Preferred FoR (in % of persons), adopted in at least 4 out of the 6 items of a set**.

**FoR**	**German**		**English**		**Chinese**		**Tongan**	
	**Non-oriented**	**Oriented**	**Non-oriented**	**Oriented**	**Non-oriented**	**Oriented**	**Non-oriented**	**Oriented**
**FRONTAL ITEMS**								
Intrinsic	—	1.4	—	3.0	—	14.3	—	9.3
Translation	5.8	8.7	22.7	22.4	**38.9**	**42.9**	**57.7**	**25.9**
Reflection	**88.4**	**84.1**	**71.2**	**64.2**	16.7	11.4	7.7	3.7
Rotation	0.0	0.0	1.5	0.0	8.3	0.0	7.7	1.9
No preference	5.8	5.8	4.5	10.4	36.1	31.4	26.9	59.3
*N*	69		66	67	36	35	52	54
**DORSAL ITEMS**								
Intrinsic	—	0.0	—	7.1	—	21.2	—	13.6
Turn-translation	0.0	0.0	0.0	0.0	3.1	0.0	1.7	0.0
Turn-reflection	8.8	7.4	1.4	0.0	3.1	6.1	1.7	1.7
Turn-rotation	**88.2**	**88.2**	**90.0**	**74.3**	**78.1**	**60.6**	**47.5**	**27.1**
No preference	2.9	4.4	8.6	18.6	15.6	12.1	49.2	57.6
*N*	68		70		32	33	59	

*Modal response printed in bold face*.

Taken together, these analyses provide information about the variability in referencing with regard to two different aspects: *individual consistency* in FoR choice and inter-individual or *cultural homogeneity*. For the four languages under scrutiny, we found different patterns. Among the German participants, high individual consistency was paired with cultural homogeneity. Almost all participants applied the same variant of the relative FoR repeatedly for the whole set of items, and most participants adopted the same variant as everybody else. However, high individual consistency need not be paired with strong cultural homogeneity. The US participants were also very consistent in applying their preferred FoR, but did not agree with each other on the frontal items regarding which type of projection to use: reflection or translation. Finally, among the Chinese and Tongan participants lower intra-individual consistency was paired with weaker cultural homogeneity: translation prevailed for frontal references and turn-rotation for dorsal references, but other variants were also applied, yet with lower consistency.

In order to further explore to which extent the possibility of adopting the intrinsic FoR (in items with an oriented ground object) contributes to lower consistency values, we cross-tabulated the preferred FoRs from the set of oriented and the set of non-oriented items. For all participants with a preference for a variant of the relative FoR on the non-oriented items we then counted how often they *kept the same* variant on the oriented items, or *switched to a different variant* of the relative FoR, to the *intrinsic* FoR, or to the *no preference* category. The results are presented in Table 6.

**Table 6:** **Keeping vs. switching the preferred FoR variant (in %) from non-oriented to oriented tasks**.

**Choice of preferred FoR**	**German**	**English**	**Chinese**	**Tongan**	**Mean**
**FRONTAL ITEMS**					
Keep relative variant	92.3	84.1	72.7	44.7	77.2
Switch to different relative variant	1.5	4.8	0.0	0.0	2.1
Switch to intrinsic FoR	1.5	3.2	18.2	10.5	5.9
Switch to “no preference”	4.6	7.9	9.1	44.7	14.4
*N*	65	63	22	38	188
**DORSAL ITEMS**					
Keep relative variant	97.0	78.1	70.4	50.0	79.1
Switch to different relative variant	0.0	0.0	3.7	0.0	0.5
Switch to intrinsic FoR	0.0	6.2	22.2	23.3	9.1
Switch to “no preference”	3.0	15.6	3.7	26.7	11.2
*N*	66	64	27	30	187

*Included are only those participants from Table 5 who preferred a relative FoR on the non-oriented tasks*.

People with an identifiable preference for a relative FoR on the non-oriented items hardly ever switched to a different variant of the relative FoR on the oriented items. They mostly kept the same variant, or switched to the intrinsic FoR (most frequently in China and Tonga), or showed no clear preference anymore (particularly in Tonga). A log-linear analysis (Kennedy, 1992) with the two factors *perspective* (frontal vs. dorsal) and *language* confirmed differences between the languages [*G*^2^(9) = 78.6; *p* < 0.001], but revealed no effect of perspective [*G*^2^(3) = 3.83; *p* = 0.280] and no interaction [*G*^2^(9) = 11.1; *p* = 0.270].

Taken together, it may be concluded that the variety of strategies in German and, more so, in English most likely reflects inter-individually different, but individually stable preferences for particular types of references. In Chinese and particularly in Tongan, however, the variety of strategies that we observed on the aggregate level seems to have two sources: Whereas many participants showed stable preferences, but differed from one another in the FoRs they adopted, others changed their referencing strategy task-specifically, which reduces their overall individual consistency.

## Discussion

The prime goal of this study was to examine the wide-spread default assumptions that people have a natural preference for the reflection variant of the relative FoR in frontal settings *(Canonical Encounter Hypothesis*; e.g., Clark, 1973), and that, in the dorsal case, they (mentally) turn around to the objects and apply the FoR which they prefer for frontal settings *(Turn Hypothesis*; e.g., Grabowski and Miller, 2000). Another, equally important goal was to introduce multinomial processing tree (MPT) models as a means to address the complexities of data collected in this field of research. Each of these goals will be discussed further in the following sections. In addition, we return to the unaccounted “unknown types of reference,” which were observed with an increased rate in the Tongan sample as compared to the other samples, and we discuss some possible reasons for it. Finally, we will take up the broader issue of what our results tell us about the relation between language, communication, and culture.

### Frontal references, the canonical encounter hypothesis, and the link between space and time

The data from the frontal configurations clearly disprove the hypothesis on the cross-cultural predominance of the reflection variant of the relative FoR: Reflection is preferred in only two of the four languages under scrutiny (German and US-English), and in one of those (US-English) not even unanimously. In Chinese and Tongan, on the other hand, reflection is relegated to the second rank by translation. Interestingly, even rotation, for which no previous cases had been reported, occurred in China and Tonga to a small, but considerable extent.

One source for variability across individuals and configurations was the absence or presence of an oriented ground object (affording an intrinsic FoR), but even beyond this specific case, a substantial number of speakers in three of the four investigated languages exhibited substantial flexibility in adopting different FoRs. This may reflect the lack of a default interpretation as described by Bohnemeyer (2011) for the Yucatec Maya and may be reinforced by a culturally encouraged inclination to take others' (or simply other) perspectives, as attested to in China and Tonga (Wu and Keysar, 2007; Beller et al., 2009; Bender et al., 2012b). Both this variability and its possible sources speak against the assumption of a “natural” preference for any particular variant of the relative FoR. Rather, the observed preferences appear to be a matter of individual proclivity, combined, to a certain extent, with linguistic and/or cultural conventions.

Please recall that the three variants of the relative FoR under scrutiny differ only in how the primary coordinate system anchored in the observer is projected onto the ground object (Figures 2, 3). The ways in which this can be done may differ in complexity with rotation requiring arguably more cognitive effort than the other two. Adopting the listener's perspective by rotation—as in a true canonical encounter—involves not only a switch on the front-back axis, but also one on the left-right axis (see Grabowski, 1999a,b). Apart from this potential difference in complexity, however, there is no *a priori* reason for considering one type of projection more appropriate than the other or for predicting a specific choice by one group of speakers compared to another. Eventually, the decision for any variant of the relative FoR is arbitrary. Once made, however, consensus among speakers would serve to facilitate communication and would therefore help to establish or maintain cultural conventions on this specific variant.

Beyond the empirical evaluation of default assumptions concerning the relative FoR, our findings also address methodological and theoretical caveats. The observation that translation is not at all rare in cross-linguistic perspective, and not even among US participants, calls for more care in theorizing and operationalization of the relative FoR. For instance, it should caution us against assuming the reflection variant as the baseline for assessing language comprehension in child development or in aphasic patients (see also Abkarian, 1982), let alone for research on spatial referencing.

Diverging preferences for reflection vs. translation may also have cognitive implications for other domains. If one presumes, for instance, a close conceptual link between the domains of space and time, the different variants of the relative spatial FoR can be assumed to also affect the relative FoR in time. And indeed, the four patterns diagnosed for temporal references map nicely on the absolute and intrinsic FoR for binary relations, and on the reflection and translation variant of the relative FoR for ternary relations (Bender and Beller, 2014; and see Bender et al., 2010). The latter type of ternary relations (i.e., those between three entities: figure, ground, and observer) are at the same time those that presuppose a distinction between future references and past references. Future events are typically located “ahead” (thus corresponding to *frontal* configurations), whereas past events are “left behind” (corresponding to *dorsal* configurations). For any attempt to relate referencing patterns across domains, taking into account the different variants of the relative FoR and the observed patterns for dorsal configurations thus proves to be indispensable (Bender et al., 2012a). Put simply: If we want to assess the extent to which preferences for spatial and temporal FoRs are related to each other, we need to know how people refer to configurations in their back (spatially) and in the past (temporally). However, recent evidence suggests that the relation between spatial and temporal FoRs is more complex, thus precluding a one-to-one mapping in language (e.g., Bender et al., 2012a; Le Guen and Pool Balam, 2012; Rothe-Wulf et al., 2015).

### Dorsal references, the turn hypothesis, and the puzzle of the turn-rotation response

In spite of the diversity in frontal tasks, most participants in our study converged on the very same response in the dorsal tasks: turn-rotation. This finding came as a great surprise. It does not only partly prove wrong the Turn Hypothesis (Grabowski and Miller, 2000), according to which people mentally turn the observer toward the objects in his or her back and then apply the relative FoR variant that they prefer for *frontal* settings; it also raises a question: Why would people, who disregard simple rotation for frontal settings, use a strategy with double rotation for dorsal ones? This is particularly puzzling if one considers that mental rotation comes with substantial cognitive costs (Shepard and Cooper, 1982; Duran et al., 2011).

**Figure 7:**
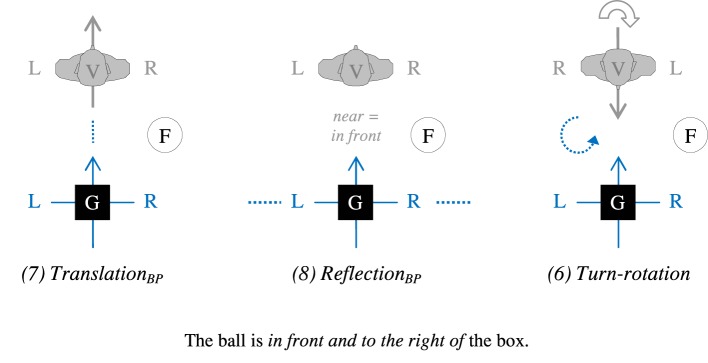
**Three strategies for dorsal references that all result in the same response (with BP, backward projection)**.

To solve this puzzle, we draw on the insight that a turn-rotation *response* might result from other strategies than turning the observer and using rotation as projection to establish a FoR. Consider a person with a preference for translation in frontal settings. When confronted with a dorsal setting, he or she could generalize this preference by simply projecting the observer's coordinate system *backwards* [Figure 7(7)]. The same holds for a person with a preference for reflection in frontal settings: When confronted with a dorsal setting, he or she could also generalize this preference by using reflection *backwards* in a retrospect way “with eyes in the back of one's head”: The space between observer and ground object G would then be regarded as near or “in front of G,” and the space beyond G as further away or “behind G” [Figure 7(8)]. Please note that this kind of reflection is not a simple mirroring of the observer's own coordinate system in the ground object in his or her back, as in that case, FRONT would point away from the observer. With FRONT always pointing *toward* the observer, and LEFT/RIGHT being taken from the observer, this FoR variant emphasizes *proximity* to the observer (or the observer's “catchment area,” as Grabowski, 1999b, p. 354 puts it). Assuming such a *backward projection* of FoRs, a turn of the *observer* is no longer necessary for a consistent transfer of frontal preferences to dorsal settings.

One possible explanation for the lack of turns is that, as already pointed out above, rotation adds cognitive complexity. Turning toward a dorsal array is inevitable only, if a person has no information about the spatial array in his or her back. If such information is available (in our case: due to visual access to the situation in the observer's back), it should be easier to start from the coordinate system as anchored in the observer and to perform a backward projection according to the same principles that apply to frontal situations.

This reinterpretation of the observed response pattern does not imply, however, that perspective taking is not involved in the production and/or comprehension of spatial descriptions in general, or that it was not involved in the tasks of our questionnaire. On the contrary: For most of our items with an oriented ground object, referencing definitely requires to take the “perspective” of the ground object, and a substantial proportion of participants did adopt the respective intrinsic FoR.

The dorsal settings and their implementation in this study raise two additional methodological questions. First, how can we identify the specific FoR a person adopts in the dorsal case, if three (hidden) strategies for dorsal references collapse to produce the same (overt) response? While logically indistinguishable, it is reasonable to consult a person's preference for frontal references in order to disentangle the different strategies. Since, for example, German speaking participants prefer the reflection variant of the relative FoR in small-scale, static, frontal settings, the “turn-rotation” response in dorsal settings results most likely from adopting the backwards projection reflection_BP_ rather than from translation_BP_ or turn-rotation. What would be more conclusive, however, is a within-subject assessment of references for frontal and dorsal configurations. This would allow one to directly relate frontal and dorsal strategies for each individual (for an application and critical evaluation of this strategy, see Beller et al., in press).

A second methodological question concerns ecological validity. Our configurations were presented as 2D sketches from a bird's eye view. Would the preferences for dorsal configurations persist when assessing them with other methods and in encounters with real objects in real space? While it is difficult to construct an alternative that does not confound the properties of the spatial layout with aspects of motion or memory—after all, people need to find out about the situation at their back before they are able to describe it—we cannot exclude that the 2D presentation may have affected our participants' dorsal responses. For the following three reasons, however, we believe that this is not the case: First, as argued previously, the dorsal data *do* indicate that participants took the perspective of the observer into account (with the objects arranged in his or her back). Second, most of their responses can be interpreted as being consistent with the preferences we observed in the frontal settings, as argued in this section. And, finally, in a recent study with German participants we were able to replicate the results for both frontal and dorsal settings with perspectival photographs as stimuli (Beller et al., in press).

### Unaccounted strategies

The category “unknown types of reference” comprised participants, whose responses are not covered by the types of FoRs discussed so far. As reported in the Results, the proportion of these responses varied between languages (German: 4.5%, English: 6.0%, Chinese: 13.7%, and Tongan: 27.2%; Table 1) and it co-varied negatively with intra-individual consistency, which was high for German and English (92.2 and 87.9% on average), but lower for Chinese (78.9%) and Tongan (64.1%; Table 4).

The MPT analysis suggests configurational complexity to be one important source of intricacy associated with these responses. Tasks are more or less complex and hence more or less difficult, depending on whether or not the reference requires a cognitively more demanding LEFT/RIGHT assignment in addition to a FRONT/BACK assignment. A more thorough inspection of the “unknown” types of references in these more complex tasks suggests two alternative, non-standard referencing strategies: For oriented items, some participants seem to have commingled two different sources in their reference, taking the FRONT/BACK assignment from the intrinsic orientation of the ground object and the LEFT/RIGHT assignment from the observer's perspective in a similar way as described in Bohnemeyer (2011) for Yucatec Maya. In other cases, participants seem to have simplified the task by disregarding the ground object G and locating the figure F in reference to the observer as ground object instead, or by disregarding the depicted observer and locating F in reference to themselves (EGO) and/or from their own perspective. These participants thus seem to use a direct FoR (Danziger, 2010)^5^ rather than a relative FoR.

### The benefits of multinomial processing tree (MPT) modeling

The MPT analysis proved to be a handy way to model spatial references—across speakers of four languages, different perspectives, and different sets of items. Specifically, it allowed us to estimate the probabilities with which participants adopted a specific FoR conditional on adopting a FoR at all (i.e., independent of such factors as item difficulty), to simultaneously consider other possibly relevant factors (such as perspective of the observer, orientation and animacy of the ground object, or configurational complexity of the item), and to analyse the data for cross-linguistic differences using inferential statistics.

Besides this more in-depth analysis of complex data sets, however, the greatest benefit of an MPT analysis is that it elegantly solves a fundamental methodological problem. As pointed out in the introduction, theoretically expanding the number of possible FoRs from which people presumably select increases the number of ambiguous responses. As the systematic exclusion of all potentially ambiguous configurations is out of the question, the MPT analysis proves to be highly valuable, as it affords to model spatial references for ambiguous and non-ambiguous items alike. Demonstrating that this is a worthwhile endeavor is one of the goals we hope to have achieved with our study. Furthermore, the finding that the full model does adequately describe the data supports the underlying assumption that participants' references on ambiguous and non-ambiguous items could be described by the same set of parameters.

### Language, culture, and communication

Descriptions based on a frame of reference are inherently ambiguous, especially those that require assignment of orientation like the relative and intrinsic FoR. Producing a description like “in front of the arrow” not only presupposes the idea that arrows have a FRONT assigned to them, but also a decision on which perspective should be adopted: the perspective of the arrow itself or that of a human observer. Likewise, comprehending the same utterance presupposes common ground on these very issues (Clark and Brennan, 1991), and conventions or shared preferences for particular FoRs help to establish this common ground.

Such preferences within a speech community, however, are not inherent in the meaning of words or in any language-specific feature for that matter, as has been argued elsewhere in some detail (Bender and Beller, 2014; Rothe-Wulf et al., 2015). They are a result of agreements or conventions within a speech community, and thus a cultural phenomenon. In this sense, studies on (linguistic) FoRs are always situated at the intersection of two dimensions, language and culture. But while the linguistic dimension of FoRs has been subject to a substantial amount of high quality research, their cultural dimension has been neglected to some extent.

Why exactly speech communities develop a preference for a particular FoR remains an open question (Majid et al., 2004). One explanation may be, of course, that the decision for a particular FoR is largely arbitrary in the first place. Each and every of the basic FoRs as well as each and every of the different variants of the relative FoR provides an equally valid description of an object array (Levinson, 2003), thus serving the same purpose with a mixture of benefits and drawbacks. But putting more effort into possible cultural factors behind these decisions, as was attempted in the MesoSpace project (Bohnemeyer et al., 2014) may still unearth valuable insights.

## Conclusion

In conclusion, neither of the two default assumptions withstands empirical testing: Reflection is not the “natural” variant of the relative FoR; translation was used with about the same frequency. And a turn of the observer is not a precondition for dorsal referencing; in our tasks, providing complete knowledge about the configuration, people used backward strategies that get by without a turn of the observer. Assuming correspondence with regard to the type of projection applied in frontal and dorsal settings, led us to re-conceptualize backward reflection as proximity. Variability in the variants of FoRs adopted is greater than one would expect, across and within languages, and sometimes even within individuals, and this variability has crucial implications for theories and methods in this field. While the exploration of this variability is constrained by the inevitably ambiguous nature of certain test items, MPT analyses provide an apt means to deal with this problem and to afford in-depth analyses of factors that influence how people make spatial references.

### Conflict of interest statement

The authors declare that the research was conducted in the absence of any commercial or financial relationships that could be construed as a potential conflict of interest.

## Footnotes

^1^Projection is what distinguishes a relative FoR from the direct FoR (Danziger, 2010), which is used for references based on the coordinate system still anchored in the observer (without any projection). If adopting the direct FoR, the spatial array in Figure 2 would be described as “The ball is in front and to the right *of the observer*.” The main difference between the direct FoR and a relaltive FoR therefore is whether the observer serves as the ground entity to which F is related (direct FoR) or wether the ground entity is different from the observer (relative FoR).

^2^Data collection at Pennsylvania State University was approved by the Institutional Review Board; in the other countries such approval was not requested due to the small scale and the non-critical nature of the study. The study was carried out in accordance with the ethical recommendations of the German Anthropological Association (DGV), and in line with the requirements for attaining research permission by the Government of the Kingdom of Tonga.

^3^Please note that the chosen sequencing does not imply a corresponding psychological ordering of the latent states, but merely reflects one of several equivalent formalizations of the underlying assumptions.

^4^Note that most of the tested models with restrictions are rejected in terms of null-hypothesis tests based on G^2^ (as indicated in the Supplementary Tables 3–7). Given our relatively large sample size of 5428 individual responses, such a misfit is to be expected. In fact, the power to detect a medium sized deviation (w = 0.3) of the observed frequencies from the expected frequencies is very large (>0.999) for the best fitting model in this first step (*df* = 216). We therefore do not report G^2^ and *p* -values in the following, but will return to this issue later.

^5^Such simplification strategies were most popular among our Tongan participants. These were younger than the participants from our other samples, and data collection took place during school hours, which might have contributed to the lower consistency values and to the adoption of such “short cut” strategies.
