# Supplementary material for: Turn around to have a look? Spatial referencing in dorsal vs. frontal settings in cross-linguistic comparison
Source: Front Psychol. 2015 Sep 2;6:1283. doi: 10.3389/fpsyg.2015.01283 (PMC4556973; doi:10.3389/fpsyg.2015.01283)
Supplement: Supplementary file 1 [file Materials_And_MPT_Analysis.PDF]

Supplementary Material

Turn around to have a look? Spatial referencing in dorsal versus frontal settings in cross-linguistic comparison

Sieghard Beller[1]*, Henrik Singmann[2], Lisa Hüther[3], & Andrea Bender[1]

[1]Department of Psychosocial Science, University of Bergen, N-5020 Bergen, Norway
[2]Department of Psychology, University of Zürich, CH-8050 Zürich, Switzerland
[3]Department of Psychology, University of Freiburg, D-79085 Freiburg, Germany

*** Correspondence:** Sieghard Beller, Department of Psychosocial Science, University of Bergen, Postbox 7807, N-5020 Bergen, Norway; email: sieghard.beller@uib.no

1. Instruction, Example Item, and Text Frames

In the following, we provide for each language the complete *instruction*, one *example item* showing how the items were presented, and the *text frames* used to introduce the response options for the different kinds of items. The pictures of the 24 spatial configurations used for the complete item set are shown in the next section. Together, this information is sufficient to reconstruct the complete questionnaires for each of the languages under scrutiny.

1.1. German

Instruction

In den folgenden Aufgaben blicken Sie von oben auf **eine Person** (grau gezeichnet, erkennbar sind Schultern und Kopf: 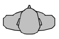 ; die Nasenspitze deutet die Blickrichtung an) und jeweils **zwei Gegenstände**, die sich im Sichtfeld dieser Person befinden. Nehmen Sie bitte die Perspektive der Person ein und bestimmen Sie, wo sich aus dieser Perspektive das weiße Objekt im Verhältnis zum schwarzen Objekt befindet. Kreuzen Sie dazu bitte wieder nur jeweils **genau eine** Antwort an!

Example Item

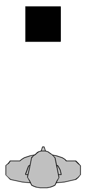

Der weiße Kreis befindet sich …

□ vor	□ links vor
□ hinter	□ rechts vor
□ links von	□ links hinter
□ rechts von	□ rechts hinter

… dem Quadrat.

Text Frames Used, Combined With the Response Options for Different Kinds of Items

Objects, non-oriented G:	Der weiße Kreis befindet sich … *{response options}* … dem Quadrat.
Living beings, non-oriented G:	Der weiße Seestern befindet sich … *{response options}* … der Blume.
Objects, oriented G:	Der weiße Kreis befindet sich … *{response options}* … dem Pfeil.
Living beings, oriented G:	Der weiße Seestern befindet sich … *{response options}* … dem Skorpion.

1.2. US-English

Instruction

In the following tasks you are looking at **a person** from above (in gray, visible are shoulders and head 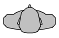 ; the tip of the nose indicates the direction of gaze) as well as **two objects** that are located in that person's visual field. Please put yourself in this person's perspective and decide where the white object is in relation to the black object from this perspective. Please check only **one answer**!

Example Item

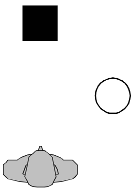

The white circle is located …

- ☐ in front of
- ☐ behind
- ☐ to the left of
- ☐ to the right of
- ☐ in front and to the left of
- ☐ in front and to the right of
- ☐ behind and to the left of
- ☐ behind and to the right of

… the square.

Text Frames Used, Combined With the Response Options for Different Kinds of Items

Objects, non-oriented G:	The white circle is located … *{response options}* … the square.
Living beings, non-oriented G:	The white starfish is located … *{response options}* … the flower.
Objects, oriented G:	The white circle is located … *{response options}* … the arrow.
Living beings, oriented G:	The white starfish is located … *{response options}* … the scorpion.

1.3. Chinese

Instruction

下图中绘有一个人头像（从上面看）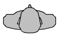

和两个物体。人头像上的鼻子指示人的目光方向。人像在看着这两个物体。请站在这个人的角度

上，你认为图上的白色物体位于黑色物体的哪个方位呢？请只选一个答案。

Example Item

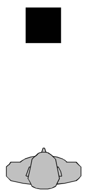

白色圆圈位于黑色方块的 …

- □ 前方
- □ 后方
- □ 左面
- □ 右面
- □ 左前方
- □ 右前方
- □ 左后方
- □ 右后方

Text Frames Used, Combined With the Response Options for Different Kinds of Items

Objects, non-oriented G: 白色圆圈位于黑色方块的 … *{response options}*
Living beings, non-oriented G: 白色海星位于花朵的 … *{response options}*
Objects, oriented G: 白色圆圈位于箭头的 … *{response options}*
Living beings, oriented G: 白色海星位于蝎子的 … *{response options}*

1.4. Tongan

Instruction

'I he ngāue ni 'oku ke vakai ai ki he tokotaha mei 'olunga ('i he lanu kulei, 'asi hake koe uma moe 'ulu: 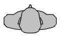 , koe mu'a ihu 'oku tuhu ki he feitu'u 'oku ne sio ki ai) pea pehē ki he me'a 'e ua 'oku tu'u 'i he feitu'u 'oku sio ki ai 'a e tokotaha ni. Kātaki 'o 'ai ko koe 'a e tokotaha ko 'eni pea ke talamai leva pē ko fe'ia 'a e me'a hinehina 'i he'ene vā moe me'a 'uli'uli. Kātaki 'o tiki'i 'a e tali pē 'e **taha** ki he fo'i fehu'i.

Example Item

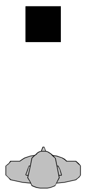

'Oku tu'u 'a e siakale hinehina …

- □ 'i mu'a he
- □ 'i mui he
- □ ki he to'ohema 'oe
- □ ki he to'omata'u 'oe
- □ 'i mu'a pea moe to'ohema 'oe
- □ 'i mu'a pea moe to'omata'u 'oe
- □ 'i mui pea moe to'ohema 'oe
- □ 'i mui pea moe to'omata'u 'oe

… koe tapafā.

Text Frames Used, Combined With the Response Options for Different Kinds of Items

Objects, non-oriented G: 'Oku tu'u 'a e siakale hinehina … *{response options}* … koe tapafā.
Living beings, non-oriented G: 'Oku tu'u 'a e mangamanga'ātai hinehina … *{response options}* … koe matala'i 'akau.
Objects, oriented G: 'Oku tu'u 'a e siakale hinehina … *{response options}* … koe tao.
Living beings, oriented G: 'Oku tu'u 'a e mangamanga'ātai hinehina … *{response options}* … koe sikopio.

2. Full List of Items and Processing Trees

The following figures present the spatial configuration of each item, together with its processing tree in the MPT analysis. The responses according to the different frames of reference (FoRs) are indicated by the abbreviations f, b, l, and r (i.e., in front of, behind, to the left of, and to the right of, respectively). The indices to the parameters indicate configurational complexity of the scene and mapping difficulty in case of an oriented item as explained in the Materials section of the article (e.g., f_3 = complexity of level 3 and i_1 = mapping difficulty of 1); lowercase letters (a, b, c) are used to distinguish parameters of the same type.

Supplementary Figure 1. Frontal items with non-oriented ground objects (black square or flower).

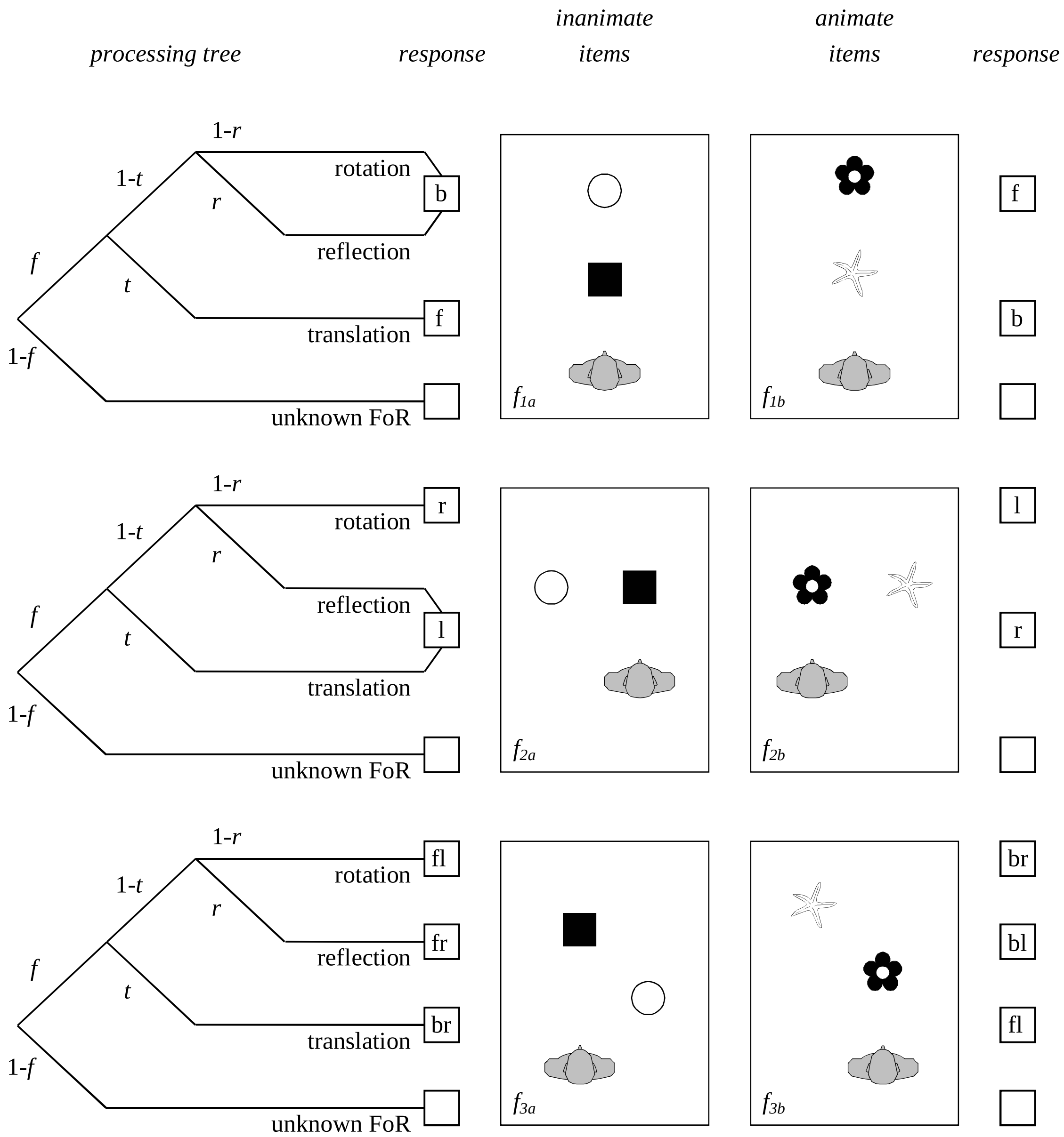

Supplementary Figure 2. Frontal items with oriented ground objects (black arrow or scorpion).

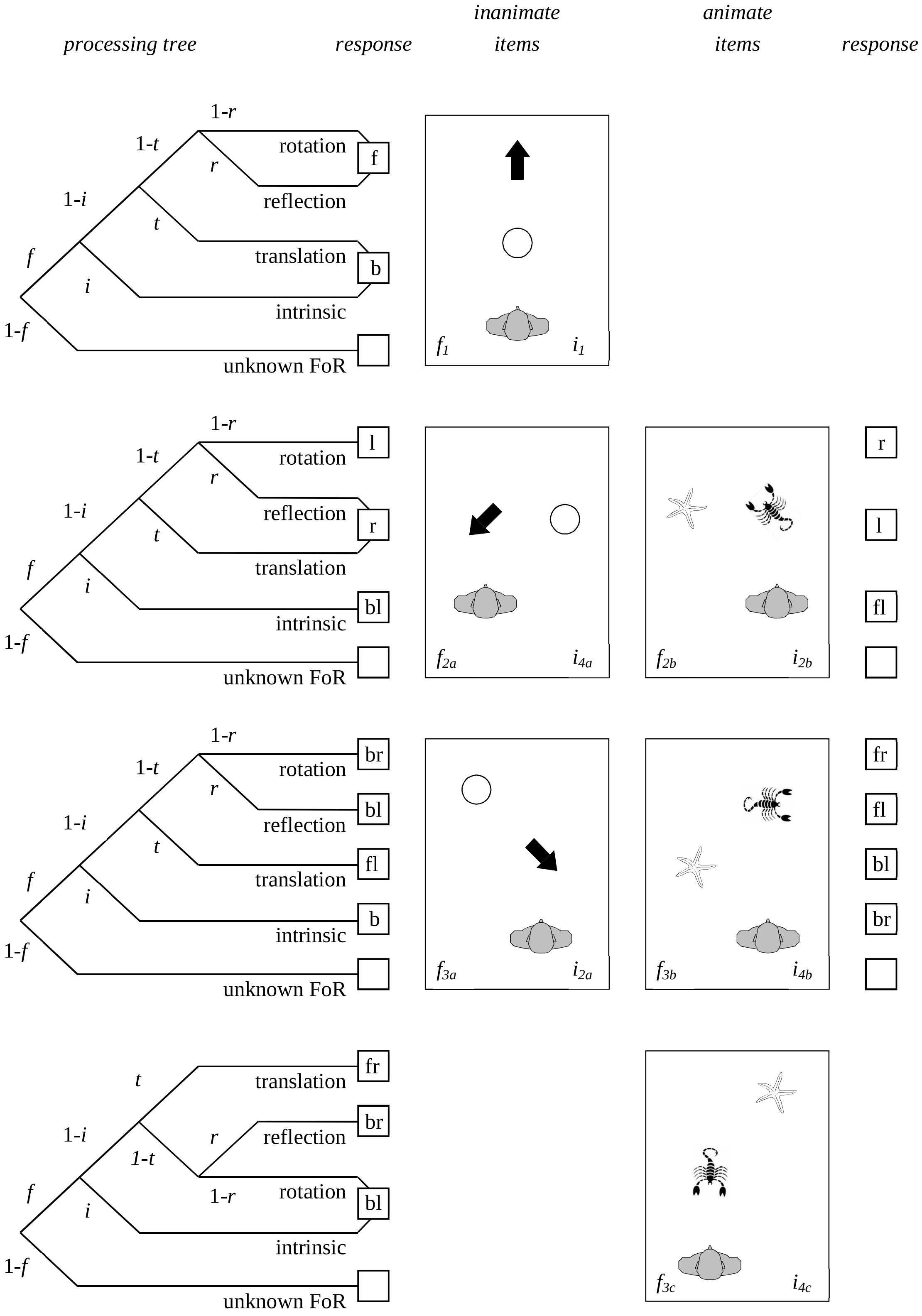

Supplementary Figure 3. Dorsal items with non-oriented ground objects (black square or flower).

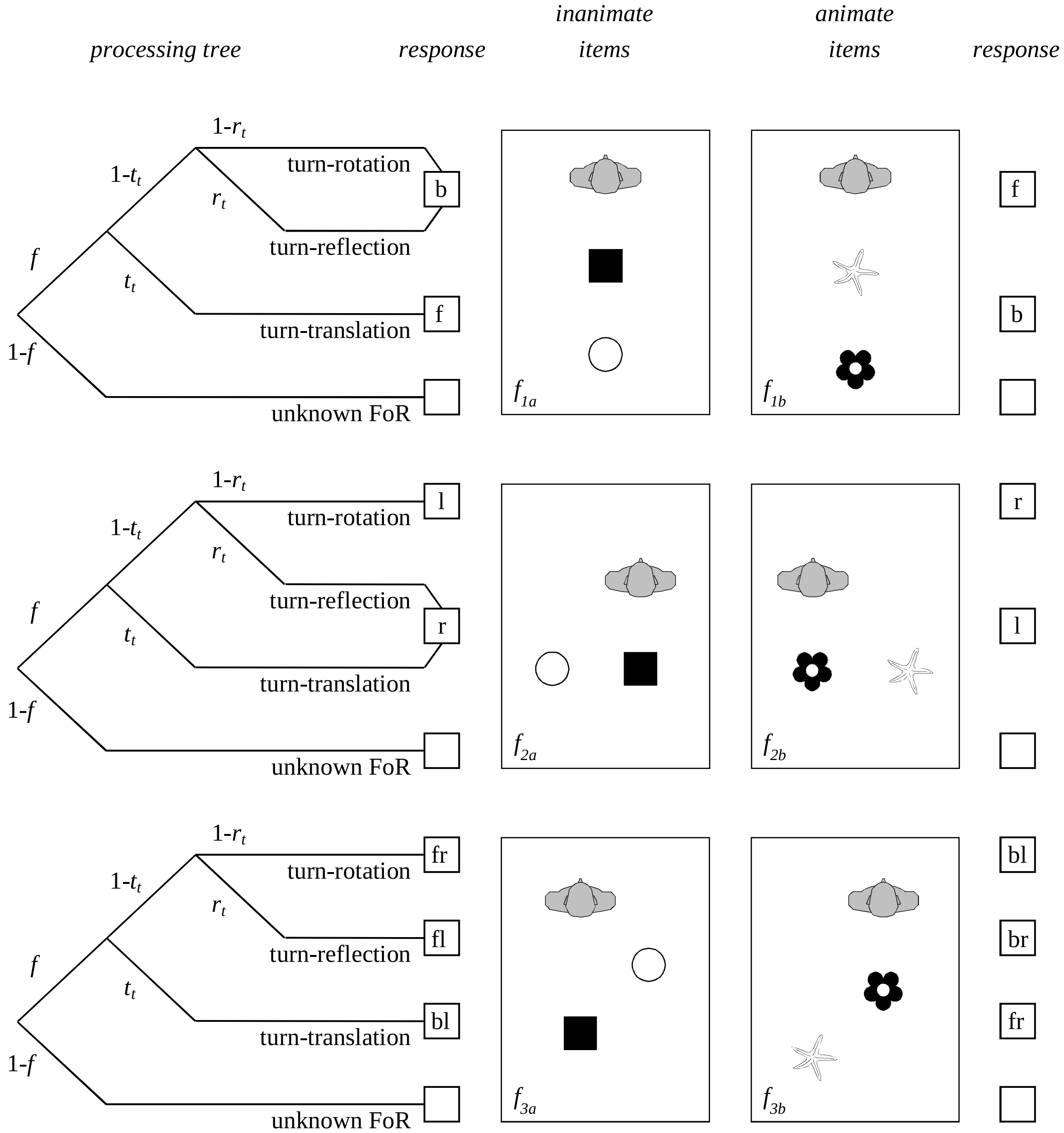

Supplementary Figure 4. Dorsal items with oriented ground objects (black arrow or scorpion).

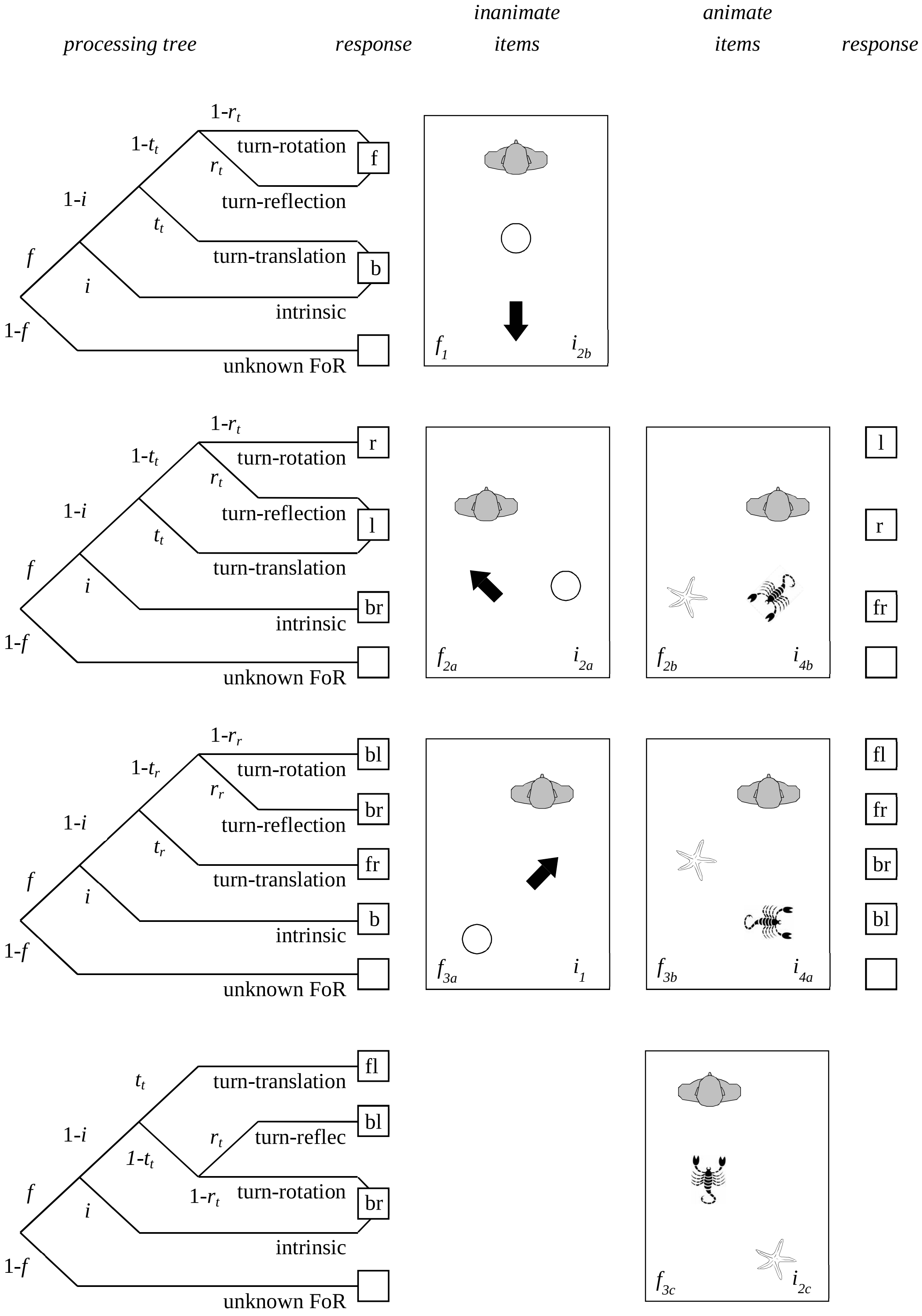

3. MPT Analysis

The following supplementary tables report the parameter estimates of the full model (section 3.1.) and all model comparisons of the model selection analysis (section 3.2.). More information including data files, processing trees and R scripts is documented in the Open Science Framework (http://osf.io/) and can be accessed through the following link:

https://osf.io/d32pk/

3.1. Parameters Estimates of the Full Model

The tables presented in this section provide the parameter estimates of the full (unrestricted) model for the frontal and the dorsal data. The confidence intervals are based on 10,000 non-parametric bootstrap samples.

Supplementary Table 1. Parameter estimates and 95% confidence intervals (in brackets) of the frontal data.

Parameter	German	English	Chinese	Tongan
(A) Frontal items with non-oriented ground object				
f_{1a}	**1.0** (1.0; 1.0)	**1.0** (1.0; 1.0)	**.89** (.78; .97)	**.92** (.85; .98)
f_{1b}	**1.0** (1.0; 1.0)	**1.0** (1.0;1.0)	**.78** (.64; .89)	**.90** (.81; .98)
f_{2a}	**.97** (.93; 1.0)	**.97** (.92; 1.0)	**.81** (.67; .92)	**.71** (.60; .83)
f_{2b}	**1.0** (1.0; 1.0)	**.97** (.92; 1.0)	**.83** (.69; .94)	**.71** (.57; .82)
f_{3a}	**.86** (.77; .93)	**.89** (.82; .97)	**.83** (.69; .94)	**.62** (.48; .75)
f_{3b}	**.87** (.78; .94)	**.92** (.86; .98)	**.81** (.67; .92)	**.62** (.48; .75)
t	**.09** (.06; .13)	**.26** (.20; .31)	**.51** (.42; .60)	**.71** (.64; .77)
r	**1.0** (.99; 1.0)	**.98** (.95; .99)	**.66** (.51; .79)	**.52** (.34; .68)
(B) Frontal items with oriented ground object				
f_1	**1.0** (1.0; 1.0)	**1.0** (1.0; 1.0)	**.83** (.69; .94)	**.96** (.91; 1.0)
f_{2a}	**.99** (.96; 1.0)	**.97** (.93; 1.0)	**.83** (.69; .94)	**.63** (.50; .76)
f_{2b}	**.99** (.96; 1.0)	**.97** (.93; 1.0)	**.86** (.74; .97)	**.76** (.65; .87)
f_{3a}	**.93** (.86; .99)	**.94** (.88; .99)	**.77** (.63; .91)	**.75** (.64; .87)
f_{3b}	**.86** (.77; .93)	**.90** (.82; .96)	**.83** (.69; .94)	**.70** (.57; .81)
f_{3c}	**.97** (.93; 1.0)	**.93** (.87; .99)	**.86** (.74; .97)	**.70** (.57; .81)
i_1	**.00** (.00; .05)	**.02** (.00; .19)	**.17** (.00; .69)	**.57** (.25; .81)
i_{2a}	**.03** (.00; .08)	**.08** (.02; .15)	**.22** (.08; .39)	**.15** (.05; .27)
$i_{2b\text{-}anim}$	**.01** (.00; .04)	**.06** (.02; .12)	**.17** (.04; .31)	**.37** (.22; .51)
i_{4a}	**.01** (.00; .04)	**.03** (.00; .08)	**.21** (.07; .37)	**.26** (.13; .42)
$i_{4b\text{-}anim}$	**.02** (.00; .05)	**.10** (.03; .18)	**.28** (.12; .45)	**.16** (.05; .28)
$i_{4c\text{-}anim}$	**.01** (.00; .06)	**.03** (.00; .08)	**.23** (.07; .41)	**.09** (.00; .25)
t	**.10** (.06; .14)	**.27** (.20; .32)	**.71** (.60; .80)	**.60** (.51; .69)
r	**.97** (.95; .99)	**.99** (.98; 1.0)	**.85** (.69; .97)	**.66** (.52; .79)

Supplementary Table 2. Parameter estimates and 95% confidence intervals (in brackets) of the dorsal data.

Parameter	German	English	Chinese	Tongan
(A) Dorsal items with non-oriented ground object				
f_{1a}	**1.0** (1.0; 1.0)	**1.0** (1.0; 1.0)	**.88** (.75; .97)	**.76** (.66; .86)
f_{1b}	**.99** (.96; 1.0)	**.97** (.93; 1.0)	**.94** (.84; 1.0)	**.78** (.66; .88)
f_{2a}	**1.0** (1.0; 1.0)	**.94** (.89; .99)	**.94** (.84; 1.0)	**.59** (.47; .71)
f_{2b}	**.99** (.96; 1.0)	**.94** (.89; .99)	**.94** (.84; 1.0)	**.63** (.51; .75)
f_{3a}	**.87** (.78; .94)	**.82** (.73; .91)	**.63** (.44; .81)	**.61** (.49; .73)
f_{3b}	**.88** (.79; .96)	**.90** (.81; .96)	**.91** (.78; 1.0)	**.75** (.63; .85)
t_t	**.03** (.01; .05)	**.04** (.02; .06)	**.09** (.05; .14)	**.14** (.09; .18)
r_t	**.08** (.05; .12)	**0.0** (0.0; 0.0)	**.04** (.00; .09)	**.05** (.01; .10)
(B) Dorsal items with oriented ground object				
f_1	**1.0** (1.0; 1.0)	**1.0** (1.0; 1.0)	**.97** (.91; 1.0)	**.86** (.76; .95)
f_{2a}	**.99** (.96; 1.0)	**.97** (.93; 1.0)	**.91** (.79; 1.0)	**.78** (.66; .88)
f_{2b}	**.96** (.90; 1.0)	**.86** (.77; .93)	**.91** (.79; 1.0)	**.49** (.37; .61)
f_{3a}	**.99** (.96; 1.0)	**.90** (.83; .96)	**.94** (.85; 1.0)	**.81** (.71; .90)
f_{3b}	**.93** (.85; .99)	**.91** (.84; .97)	**.91** (.79; 1.0)	**.75** (.63; .85)
f_{3c}	**.93** (.85; .99)	**.89** (.81; .96)	**.94** (.85; 1.0)	**.71** (.59; .81)
i_1	**.03** (.00; .07)	**.13** (.05; .21)	**.23** (.09; .38)	**.28** (.15; .41)
i_{2a}	**.01** (.00; .05)	**.12** (.04; .20)	**.30** (.14; .47)	**.43** (.29; .58)
i_{2b}	**.03** (.00; .09)	**.24** (.14; .34)	**.33** (.17; .51)	**.44** (.30; .58)
$i_{2c\text{-}anim}$	**.09** (.00; .73)	**.31** (.00; 1.0)	**.35** (.00; 1.0)	**.07** (.00; .67)
$i_{4a\text{-}anim}$	**.03** (.00; .08)	**.30** (.19; .41)	**.33** (.17; .50)	**.43** (.29; .58)
$i_{4b\text{-}anim}$	**.00** (.00; .00)	**.08** (.02; .16)	**.17** (.04; .31)	**.28** (.12; .45)
t_t	**.01** (.00; .03)	**.00** (.00; .02)	**.02** (.00; .06)	**.03** (.00; .08)
r_t	**.10** (.07; .13)	**.02** (.00; .04)	**.08** (.03; .15)	**.15** (.09; .23)

3.2. Model Selection Analysis

The tables presented in this section provide the model comparisons of the model selection analysis (steps 1 to 4). For the FIA analyses, three values are reported: the *FIA penalty* corresponds to the penalty term for a model's flexibility that is added to a model's fit value, the *FIA value* is the sum of the fit value (which is ½ G^2 for FIA) and the penalty, and *ΔFIA* corresponds to each model's FIA value minus the smallest FIA value per table (i.e., the best model in terms of FIA always has *ΔFIA* = 0).

Supplementary Table 3. FIA-analysis of the *f* and *i* parameters for the four languages simultaneously (step 1); best model printed in **bold** face.

Model	*n* parameter per language	G^2_{sum}	df_{sum}	p_{sum}	FIA-penalty$_{sum}$	FIA$_{sum}$	ΔFIA$_{sum}$
f: free & i: free	44	80.1	88	.714	360.6	400.6	127.7
f: free & i: perspectival mapping	38	115.2	112	.400	330.6	388.2	115.3
f: free & i: perspectival animacy	36	126.9	120	.316	319.6	383.1	110.2
f: free & i: global mapping	35	157.6	124	.022	312.1	390.9	118.0
f: free & i: global animacy	34	160.1	128	.029	304.9	384.9	112.0
f: free & i: all equal	33	161.6	132	.041	296.2	377.0	104.1
f: local complexity & i: free	32	138.1	136	.433	276.0	345.1	72.2
f: local complexity & i: perspectival mapping	26	173.2	160	.225	241.3	327.9	55.0
f: local complexity & i: perspectival animacy	24	184.9	168	.176	229.5	321.9	49.0
f: local complexity & i: global mapping	23	215.6	172	.013	221.5	329.3	56.4
f: local complexity & i: global animacy	22	218.2	176	.017	213.8	322.9	50.0
f: local complexity & i: all equal	21	219.6	180	.024	204.8	314.7	41.8
f: perspectival complexity & i: free	26	171.2	160	.258	227.6	313.2	40.3
f: perspectival complexity & i: perspectival mapping	20	206.3	184	.125	191.9	295.0	22.1
f: perspectival complexity & i: perspectival animacy	18	218.0	192	.096	179.6	288.6	15.7
f: perspectival complexity & i: global mapping	17	248.7	196	.006	171.6	295.9	23.0
f: perspectival complexity & i: global animacy	16	251.2	200	.008	163.6	289.2	16.3
f: perspectival complexity & i: all equal[To]	15	252.7	204	.012	154.5	280.8	7.9
f: global complexity & i: free	23	212.1	172	.020	203.1	309.2	36.3
f: global complexity & i: perspectival mapping	17	247.1	196	.008	165.0	288.6	15.7
f: global complexity & i: perspectival animacy[En]	15	258.8	204	.006	152.2	281.6	8.7
f: global complexity & i: global mapping	14	289.6	208	<.001	143.7	288.5	15.6
f: global complexity & i: global animacy	13	292.1	212	<.001	135.5	281.6	8.7
***f: global complexity & i: all equal*[Ge]**	**12**	**293.6**	**216**	**<.001**	**126.1**	**272.9**	**0.0**
f: all equal & i: free	21	385.9	180	<.001	186.1	379.0	106.1
f: all equal & i: perspectival mapping	15	421.0	204	<.001	146.3	356.8	83.9
f: all equal & i: perspectival animacy	13	432.7	212	<.001	132.2	348.5	75.6
f: all equal & i: global mapping	12	463.4	216	<.001	123.4	355.1	82.2
f: all equal & i: global animacy	11	466.0	220	<.001	114.9	347.9	75.0
f: all equal & i: all equal[Ch]	10	467.4	224	<.001	105.2	338.9	66.0

[To] Best model for Tongan ($G^2[51] = 83.5$; $p = .003$; $FIA = 80.6$); [En] best model for English ($G^2[51] = 64.4$; $p = .099$; $FIA = 71.9$);
[Ge] Best model for German ($G^2[54] = 50.0$; $p = .629$; $FIA = 57.9$); [Ch] best model in Chinese ($G^2[56] = 57.7$; $p = .414$; $FIA = 52.7$).

Supplementary Table 4. FIA-analysis of the *t*, *r*, t_t and r_t parameters for the four languages simultaneously (step 2, starting from the best model of step 1, Supplementary Table 3); best model printed in **bold** face.

Model	*n* parameter per language	G^2_{sum}	df_{sum}	p_{sum}	FIA-penalty$_{sum}$	FIA$_{sum}$	ΔFIA$_{sum}$
t: local & r: local & t_t: local & r_t: local	12	293.6	216	<.001	126.2	272.9	10.5
t: global & r: local & t_t: local & r_t: local	11	303.0	220	<.001	117.5	269.0	6.6
t: local & r: global & t_t: local & r_t: local	11	305.6	220	<.001	119.0	271.7	9.3
t: global & r: global & t_t: local & r_t: local[To]	10	316.6	224	<.001	109.2	267.5	5.1
t: local & r: local & t_t: global & r_t: local	11	309.9	220	<.001	117.5	272.4	10.0
t: global & r: local & t_t: global & r_t: local	10	319.4	224	<.001	109.0	268.7	6.3
t: local & r: global & t_t: global & r_t: local	10	321.8	224	<.001	110.5	271.4	8.9
t: global & r: global & t_t: global & r_t: local	9	332.9	228	<.001	100.9	267.3	4.9
t: local & r: local & t_t: local & r_t: global	11	303.9	220	<.001	119.0	270.9	8.5
t: global & r: local & t_t: local & r_t: global	10	313.4	224	<.001	110.5	267.2	4.7
t: local & r: global & t_t: local & r_t: global	10	315.9	224	<.001	111.9	269.9	7.4
t: global & r: global & t_t: local & r_t: global	9	327.0	228	<.001	102.3	265.8	3.3
t: local & r: local & t_t: global & r_t: global[Ch]	10	315.7	224	<.001	109.2	267.0	4.6
t: global & r: local & t_t: global & r_t: global	9	325.2	228	<.001	100.8	263.4	1.0
t: local & r: global & t_t: global & r_t: global	9	327.7	228	<.001	102.3	266.1	3.7
***t: global & r: global & t_t: global & r_t: global*[Ge; En]**	**8**	**338.8**	**232**	**<.001**	**93.1**	**262.5**	**0.0**

[To] Best model for Tongan ($G^2[56] = 103.8$; p = <.001; *FIA* = 79.4); [Ch] Best model for Chinese ($G^2[56] = 61.5$; $p = .286$; *FIA* = 55.7); [Ge] best model for German ($G^2[58] = 56.0$; $p = .549$; *FIA* = 52.2); [En] best model for English ($G^2[58] = 93.3$; $p = .002$; *FIA* = 70.8).

Supplementary Table 5. FIA-analysis of the *f* and *i* parameters across the four languages (step 3, starting from the best model of step 2, Supplementary Table 4); best model printed in **bold** face.

Model	*n* parameter	G^2	*df*	*p*	FIA-penalty	FIA	ΔFIA
f: all languages different & i: all languages different	32	338.8	232	<.001	93.1	262.5	4.0
f: all languages different & i: non-germanic equal	31	341.4	233	<.001	90.6	261.3	2.9
f: all languages different & i: germanic equal	31	388.6	233	<.001	90.4	284.7	26.2
f: all languages different & i: g. equal+non-g. equal	30	391.3	234	<.001	88.0	283.6	25.1
f: all languages different & i: all languages equal	29	548.1	235	<.001	85.3	359.4	100.9
f: non-germanic equal & i: all languages different	29	407.2	235	<.001	85.4	289.0	30.5
f: non-germanic equal & i: non-germanic equal	28	409.8	236	<.001	82.9	287.8	29.3
f: non-germanic equal & i: germanic equal	28	457.1	236	<.001	82.7	311.2	52.7
f: non-germanic equal & i: g. equal+non-g. equal	27	459.7	237	<.001	80.3	310.1	51.6
f: non-germanic equal & i: all languages equal	26	616.5	238	<.001	77.6	385.8	127.3
f: germanic equal & i: all languages different	29	349.9	235	<.001	84.6	259.6	1.1
f: germanic equal & i: non-germanic equal	**28**	**352.6**	**236**	**<.001**	**82.2**	**258.5**	**0.0**
f: germanic equal & i: germanic equal	28	399.8	236	<.001	81.9	281.8	23.3
f: germanic equal & i: g. equal+non-g. equal	27	402.4	237	<.001	79.5	280.7	22.2
f: germanic equal & i: all languages equal	26	559.2	238	<.001	76.9	356.5	98.0
f: g. equal+non-g. equal & i: all languages different	26	418.4	238	<.001	77.0	286.1	27.7
f: g. equal+non-g. equal & i: non-germanic equal	25	421.0	239	<.001	74.5	285.0	26.5
f: g. equal+non-g. equal & i: germanic equal	25	468.2	239	<.001	74.2	308.3	49.8
f: g. equal+non-g. equal & i: g. equal+non-g. equal	24	470.9	240	<.001	71.7	307.2	48.7
f: g. equal+non-g. equal & i: all languages equal	23	627.2	241	<.001	69.1	382.9	124.4
f: all languages equal & i: all languages different	23	827.9	241	<.001	68.4	482.4	223.9
f: all languages equal & i: non-germanic equal	22	830.5	242	<.001	65.9	481.1	222.6
f: all languages equal & i: germanic equal	22	877.8	242	<.001	65.7	504.5	246.0
f: all languages equal & i: g. equal+non-g. equal	21	880.4	243	<.001	63.1	503.3	244.8
f: all languages equal & i: all languages equal	20	1037.2	244	<.001	60.3	578.9	320.5

Supplementary Table 6. FIA-analysis of the *t* and *r* parameters across the four languages (step 4 for the frontal data, starting from the best model of step 3, Supplementary Table 5); best model printed in **bold** face.

Model	*n* parameter	G^2	*df*	*p*	FIA-penalty	FIA	ΔFIA
t: all languages different & r: all languages different	28	352.6	236	<.001	82.2	258.5	3.6
t: all languages different & r: non-germanic equal	27	354.8	237	<.001	80.3	257.7	2.8
t: all languages different & r: germanic equal	27	352.6	237	<.001	80.1	256.4	1.5
t: all languages different & r: g. equal+non-g. equal	26	354.9	238	<.001	78.1	255.6	0.7
t: all languages different & r: all languages equal	25	518.3	239	<.001	75.6	334.8	79.9
t: non-germanic equal & r: all languages different	27	357.3	237	<.001	79.9	258.6	3.7
t: non-germanic equal & r: non-germanic equal	26	358.6	238	<.001	77.7	257.0	2.1
t: non-germanic equal & r: germanic equal	26	357.4	238	<.001	77.7	256.4	1.5
t: non-germanic equal & r: g. equal+non-g. equal	**25**	**358.7**	**239**	**<.001**	**75.6**	**254.9**	**0.0**
t: non-germanic equal & r: all languages equal	24	522.9	240	<.001	73.1	334.5	79.6
t: germanic equal & r: all languages different	27	397.5	237	<.001	79.6	278.4	23.5
t: germanic equal & r: non-germanic equal	26	399.7	238	<.001	77.7	277.6	22.7
t: germanic equal & r: germanic equal	26	397.7	238	<.001	77.2	276.0	21.1
t: germanic equal & r: g equal+non-g. equal	25	399.9	239	<.001	75.2	275.2	20.3
t: germanic equal & r: all languages equal	24	563.4	240	<.001	72.8	354.5	99.6
t: g. equal+non-g. equal & r: all languages different	26	402.3	238	<.001	77.3	278.5	23.6
t: g. equal+non-g. equal & r: non-germanic equal	25	403.5	239	<.001	75.1	276.9	22.0
t: g. equal+non-g. equal & r: germanic equal	25	402.5	239	<.001	74.9	276.1	21.2
t: g. equal+non-g. equal & r: g. equal+non-g. equal	24	403.7	240	<.001	72.6	274.5	19.6
t: g. equal+non-g. equal & r: all languages equal	23	567.9	241	<.001	70.3	354.2	99.3
t: all languages equal & r: all languages different	25	729.6	239	<.001	74.8	439.6	184.7
t: all languages equal & r: non-germanic equal	24	731.2	240	<.001	72.5	438.1	183.2
t: all languages equal & r: germanic equal	24	729.8	240	<.001	72.2	437.1	182.2
t: all languages equal & r: g. equal+non-g. equal	23	731.4	241	<.001	69.9	435.6	180.7
t: all languages equal & r: all languages equal	22	858.0	242	<.001	67.2	496.2	241.3

Supplementary Table 7. FIA-analysis of the t_t and r_t parameters across the four languages (step 4 for the dorsal data, starting from the best model of step 3, Supplementary Table 5); best model printed in **bold** face.

Model	*n* parameter	G^2	*df*	*p*	FIA-penalty	FIA	ΔFIA
t_t: all languages different & r_t: all languages different	28	352.6	236	<.001	82.2	258.5	3.2
t_t: all languages different & r_t: non-germanic equal	27	354.9	237	<.001	80.3	257.8	2.5
t_t: all languages different & r_t: germanic equal	27	387.7	237	<.001	80.0	273.9	18.6
t_t: all languages different & r_t: g. equal+non-g. equal	26	390.0	238	<.001	78.1	273.2	17.9
t_t: all languages different & r_t: all languages equal	25	394.4	239	<.001	75.6	272.8	17.5
t_t: non-germanic equal & r_t: all languages different	27	355.4	237	<.001	80.0	257.7	2.4
t_t: non-germanic equal & r_t: non-germanic equal	26	359.1	238	<.001	77.8	257.3	2.0
t_t: non-germanic equal & r_t: germanic equal	26	390.5	238	<.001	77.7	273.0	17.7
t_t: non-germanic equal & r_t: g. equal+non-g. equal	25	394.2	239	<.001	75.6	272.7	17.4
t_t: non-germanic equal & r_t: all languages equal	24	398.6	240	<.001	73.1	272.4	17.2
t_t: germanic equal & r_t: all languages different	27	353.6	237	<.001	79.7	256.5	1.2
t_t: germanic equal & r_t: non-germanic equal	26	356.0	238	<.001	77.8	255.8	0.5
t_t: germanic equal & r_t: germanic equal	26	388.0	238	<.001	77.1	271.1	15.8
t_t: germanic equal & r_t: g. equal+non-g. equal	25	390.4	239	<.001	75.2	270.4	15.1
t_t: germanic equal & r_t: all languages equal	24	394.6	240	<.001	72.8	270.1	14.8
t_t: g. equal+non-g. equal & r_t: all languages different	26	356.5	238	<.001	77.4	255.6	0.3
t_t: g. equal+non-g. equal & r_t: non-germanic equal	**25**	**360.1**	**239**	**<.001**	**75.2**	**255.3**	**0.0**
t_t: g. equal+non-g. equal & r_t: germanic equal	25	390.8	239	<.001	74.9	270.3	15.0
t_t: g. equal+non-g. equal & r_t: g. equal+non-g. equal	24	394.5	240	<.001	72.7	269.9	14.6
t_t: g. equal+non-g. equal & r_t: all languages equal	23	398.8	241	<.001	70.3	269.7	14.4
t_t: all languages equal & r_t: all languages different	25	383.8	239	<.001	74.8	266.7	11.5
t_t: all languages equal & r_t: non-germanic equal	24	387.6	240	<.001	72.5	266.3	11.1
t_t: all languages equal & r_t: germanic equal	24	419.9	240	<.001	72.2	282.1	26.8
t_t: all languages equal & r_t: g. equal+non-g. equal	23	423.7	241	<.001	69.9	281.8	26.5
t_t: all languages equal & r_t: all languages equal	22	435.5	242	<.001	67.2	285.0	29.7
